# Dynamical selection of Nash equilibria using reinforcement learning: Emergence of heterogeneous mixed equilibria

**DOI:** 10.1371/journal.pone.0196577

**Published:** 2018-07-09

**Authors:** Robin Nicole, Peter Sollich

**Affiliations:** Department of Mathematics, King’s College London, Strand, London, WC2R 2LS, United Kingdom; Universidad Veracruzana, MEXICO

## Abstract

We study the distribution of strategies in a large game that models how agents choose among different double auction markets. We classify the possible mean field Nash equilibria, which include potentially segregated states where an agent population can split into subpopulations adopting different strategies. As the game is aggregative, the actual equilibrium strategy distributions remain undetermined, however. We therefore compare with the results of a reinforcement learning dynamics inspired by Experience-Weighted Attraction (EWA) learning, which at long times leads to Nash equilibria in the appropriate limits of large intensity of choice, low noise (long agent memory) and perfect imputation of missing scores (fictitious play). The learning dynamics breaks the indeterminacy of the Nash equilibria. Non-trivially, depending on how the relevant limits are taken, more than one type of equilibrium can be selected. These include the standard homogeneous mixed and heterogeneous pure states, but also *heterogeneous mixed* states where different agents play different strategies that are not all pure. The analysis of the reinforcement learning involves Fokker-Planck modeling combined with large deviation methods. The theoretical results are confirmed by multi-agent simulations.

## Introduction

Agent based models describe the dynamics of co-learning and interacting individuals and can be applied in many fields including sociology—with the Schelling model of segregation [1] a famous example—and economics, where the individuals are economic agents. In recent decades, there has been growing interest in the application of agent based models to the study of financial markets; for extensive reviews of such applications we refer to [2, 3]. Among existing models of double auction markets, one can cite the work of Iori et al. [4] and the CAT game [5]. The latter is a market design tournament in which participants were asked to supply automated markets that would perform as well as possible in an economic system populated with automated traders. Spontaneous emergence of preferences for different markets emerged within the population of traders. Unfortunately, the complexity of the CAT game tournament made it impossible to study this phenomenon by analytical methods, emphasizing the need for a simpler model to understand it. Alorić et al. designed such a minimal version of the CAT game, where traders learn to choose among *two* double auction markets [6]. Also there spontaneous emergence of preferences heterogeneity was observed, as the outcome of the learning dynamics. Whether this result has an interpretation as a game theoretical equilibrium was not addressed, however. This will be one of the two main questions of this paper: we ask to what extent this spontaneous emergence of preferences heterogeneity shows up in the *Nash equilibria* of the game corresponding to the model of Alorić *et al*.. One of the properties of this game is that the payoff agents earn by trading at the different markets depends only on the ratio of the number of buyers and sellers at this market. The game therefore belongs to the class of aggregative games, where payoffs depend on a finite number of macroscopic quantities, called aggregates.

Bearing in mind the above broader context, we consider in this paper the double auction game of [6] as a paradigmatic example of an aggregative game with an infinitely large number of players. While it is known that finding Nash equilibria in games with a large but finite number of players is computationally hard [7], taking the number of players to infinity can lead to drastic simplifications that make the problem analytically tractable. This is because the limit eliminates some features such as the market impact of the action of a single player [8]. For aggregative games the limit also has convenient mathematical properties: Nash equilibria of infinite games can be characterized as the large size limit of equilibria in games with a finite number of players [9]. An introduction to games with a large number of players would not be complete without mentioning mean field game theory [10, 11], which studies stochastic differential games with an infinite number of players. The underlying formalism here is rather different from the one we use in the rest of this article, however.

Nash equilibria of aggregative game are characterized by the values of the aggregates on which the payoff of any given action depends. To each of these there generally correspond infinitely many different distributions of strategies among the players. In this paper, the second question we therefore ask is whether and how this degeneracy in the strategy distribution is resolved by the learning dynamics of the corresponding agent based model. This issue of how a Nash equilibrium is selected dynamically has been studied theoretically for games of small size [12] and using numerical simulation for larger games [13–15], providing results on the speed of convergence and efficiency of certain types of learning dynamics. While these previous studies focused on the value of macroscopic quantities such as the ratio of number of buyers to number of sellers once the learning dynamics has converged, we are interested in going further and investigating the distribution of strategies, which is crucial in order to establish whether the distribution of preferences of traders is multimodal or not. Although there are many studies on the convergence of standard learning dynamics such as *fictitious play* to Nash equilibria [12, 16, 17] and study of the basin of attraction of such equilibria under different learning dynamics [18]; studies on the emergence of preferences heterogeneity as the outcome of a learning dynamics remain absent from the literature. The specific learning rule we study a form of reinforcement learning inspired by Experience Weighted Attraction (EWA) learning, which is well known to reproduce quite accurately the behaviour of human subjects learning to play repeated normal form games [19]. Strategies are encoded by so-called preferences in this approach, and the comparison of the *preference distributions* that result from reinforcement learning dynamics with the properties of the underlying Nash equilibria is one of our main contributions; this is a novel approach that has not to our knowledge been pursued in the existing literature.

Methodically, we argue that in the game we analyse, correspondence with Nash equilibria requires a long memory limit. The reinforcement learning dynamics of the agents is then described by a Fokker-Planck equation, and it is the steady states of this that we study. We deploy large deviation methods to detect multimodality in the preferences distribution, where agents split into sub-populations that each play a different strategy. We combine this approach with numerical simulations in order to shed light on the several, qualitatively different, types of preference distribution that can emerge in the steady state of the learning dynamics. These include the two scenarios that are conventionally considered: homogeneous mixed equilibria, where all agents play the same mixed strategy, and heterogeneous pure equilibria, where different agents play different pure strategies [20–22]. Surprisingly, however, we also find heterogeneous mixed solutions, where the agents play different strategies and these strategies themselves include mixed strategies.

This paper is organized as follow. In the model section, we summarize the minimal model of traders choosing between double auction markets to be studied in the rest of this article, as well as the variation on EWA learning dynamics we use. In the result section, we study the Nash equilibria of the aggregative game corresponding to this model, in the limit of a large number of players. In in the results section starting we present a study of the steady states of the learning dynamics in the model presented in page 3 and argue that in the limit of *fictitious play*, *best response dynamics* and *large memory*, these steady states are Nash equilibria. We show that depending on how these multiple limits are approached, the dynamics selects several distinct Nash equilibria, including ones of heterogeneous mixed type. In the method sections, we present separately the large deviation methods that we use in our study of the steady states of the reinforcement learning model in the large memory limit. In the conclusion summarizes our results and lays out some avenues for future research.

## Model: Choosing between double auction markets

In this section, we summarize the model of double auction markets of Alorić *et al*. [6]. In this model, a population of co-evolving traders competes to trade by choosing between two double auction markets. This can lead to spontaneous emergence of heterogeneous preferences, where agents spontaneously split into groups with different preferences for the two markets. The model contains three ingredients: (i) the market mechanism by which the double auction markets process orders to buy and sell, (ii) the way traders set their order prices (this is assumed fixed and not affected by learning) and calculate their payoff, and (iii) the learning procedure that traders use to learn their trading strategy, i.e. their preference for each market. We describe these three ingredients in turn.

### Market mechanism

The model assumes that each market processes orders in discrete trading rounds rather than continuously. In each round each trader places at one of the markets an order to buy or sell one unit of the underlying good. An order is denoted (*τ*, *p*) where *τ* ∈ {a, b} designates the type of order, with a an order to sell (also known as an ask) and b an order to buy (a bid); *p* is the price at which the trader proposes to buy or sell. For example (b, 20) is an order to buy one unit of good at a price of 20. Once all the traders have sent their orders (see Dynamics of traders), the clearing process begins. The trading price is set by each market using the formula

$$\begin{array}{c} \pi_{m}=\left(1-\theta_{m}\right)\left\langle \text{b}\right\rangle +\theta_{m}\left\langle \text{a}\right\rangle \end{array}$$ (1)

where 〈b〉, 〈a〉 are the average prices of bids and asks received by the market. All the orders on the wrong side of the trading price (*i.e*. an order to buy lower than the trading price or an order to sell higher than the trading price) are rejected. The remaining *valid orders* are *executed* at the trading price by randomly forming pairs of one buyer and one seller until no more pairs can be formed. As the number of valid bids and asks will differ in general, some traders will remain unmatched; they are unable to trade and their orders are not executed.

### Order pricing and payoff calculation

As explained above, it is assumed that traders *always* send an order to buy or sell *exactly* one unit of good to only one single market. This is done to keep the model as simple as possible. Following the work of Gode and Sunders [23], traders set the price of their orders with *zero intelligence*: the price of each order to buy (resp. sell) sent by each trader is an independent Gaussian random variable with mean *μ*_b_ (resp. *μ*_a_) and standard deviation *σ*_b_ = *σ*_a_ = 1. While this assumption may appear drastic at first sight, Gode and Sunders found that traders sending orders to double auction markets with zero intelligence was a good substitute for individual rationality [23]. The model also assumes that each agent chooses randomly whether to buy or sell, with a fixed probability *p*_b_ that can be different for different agents.

At the end of a trading round, each trader receives as feedback from the market to which they sent their order whether it was executed and if so at which price. From this each trader computes the score of his order S as either zero, if the order was not executed, or otherwise as the profit of the order, which in the model is defined as the absolute value of the difference between order price and trading price. This payoff is random and is affected by: (i) the submitted order price, (ii) the trading price, and (iii) whether the order is executed, which in turn depends on the ratio of number of buyers and sellers in the market where the offer was sent. (We discuss in the results section how the average payoff over these sources of randomness can be calculated in the limit of a large system.)

### Dynamics of traders

The remaining part of the behaviour of the traders that the model needs to prescribe is how they learn their respective preferences for the two markets. The assumption is that agents use a variation of experience-weighted attraction reinforcement learning (EWA) [19]. They have attractions *A*_*m*_ to each market *m* ∈ {1, 2}, which they update after each trading round *n* according to

$$\begin{array}{c} A_{m}\left(n+1\right)=\left\{ \begin{array}{cc} \left(1-r\right)A_{m}\left(n\right)+rS\left(n\right) & \text{if}\;\text{the}\;\text{agent}\;\text{chose}\;\text{market}\;m\;\text{in}\;\text{round}\;n\\ \left(1-\alpha r\right)A_{m}\left(n\right) & \text{otherwise} \end{array}\right. \end{array}$$ (2)

Here S(n) is the payoff for the order placed at time-step *n*, *α* is a *fictitious play parameter* which describes how fast traders decrease the attraction to actions they do not play, and *r* is the inverse of the agents’ memory, defined as the period of time over which they typically remember past payoffs. Based on those attractions **A** = (*A*_1_, *A*_2_), traders then randomly choose a market for trade according to the inverse logit or “softmax” function *σ*_*β*_(⋅),

$$\begin{array}{c} P\left(\text{trade}\;\text{at}\;\text{market}\;1|A\right)=\sigma_{\beta }\left(A_{1}-A_{2}\right)=\frac{1}{1+\exp(-\beta \left(A_{1}-A_{2}\right))} \end{array}$$ (3)

where *β* is the intensity of choice that regulates how strongly the agents use the attractions to bias their preferences. Note that in the equation above, traders update their attraction to the market they did not choose using only their attraction to this market and not its payoff as is the case in EWA learning dynamics described in Ref. [19] and in stochastic fictitious play [16, 17]. The reason for this choice is that in our model, traders do not have information about the payoff in the market they did not trade, so we effectively replace this unknown payoff by (1 − *α*)*A*_*m*_(*t*). This absence of information about the action they did not play is one of the reasons why traders end up with heterogeneous preferences.

A possible extension of this setup, which we do not pursue here, is to allow the traders to learn also their preference for buying and selling, instead of keeping this fixed [6]. In that case there would be four attractions to be learned, for buying and selling at each of the two markets.

We shall use “reinforcement learning model” as a shorthand to designate the above dynamics where traders learn at which market to trade—note that because of this learning process the traders are somewhat more intelligent than the strictly zero-intelligence traders described by Gode and Sunders [23], who in our scenario would choose randomly also where to trade.

**Fig 1:**
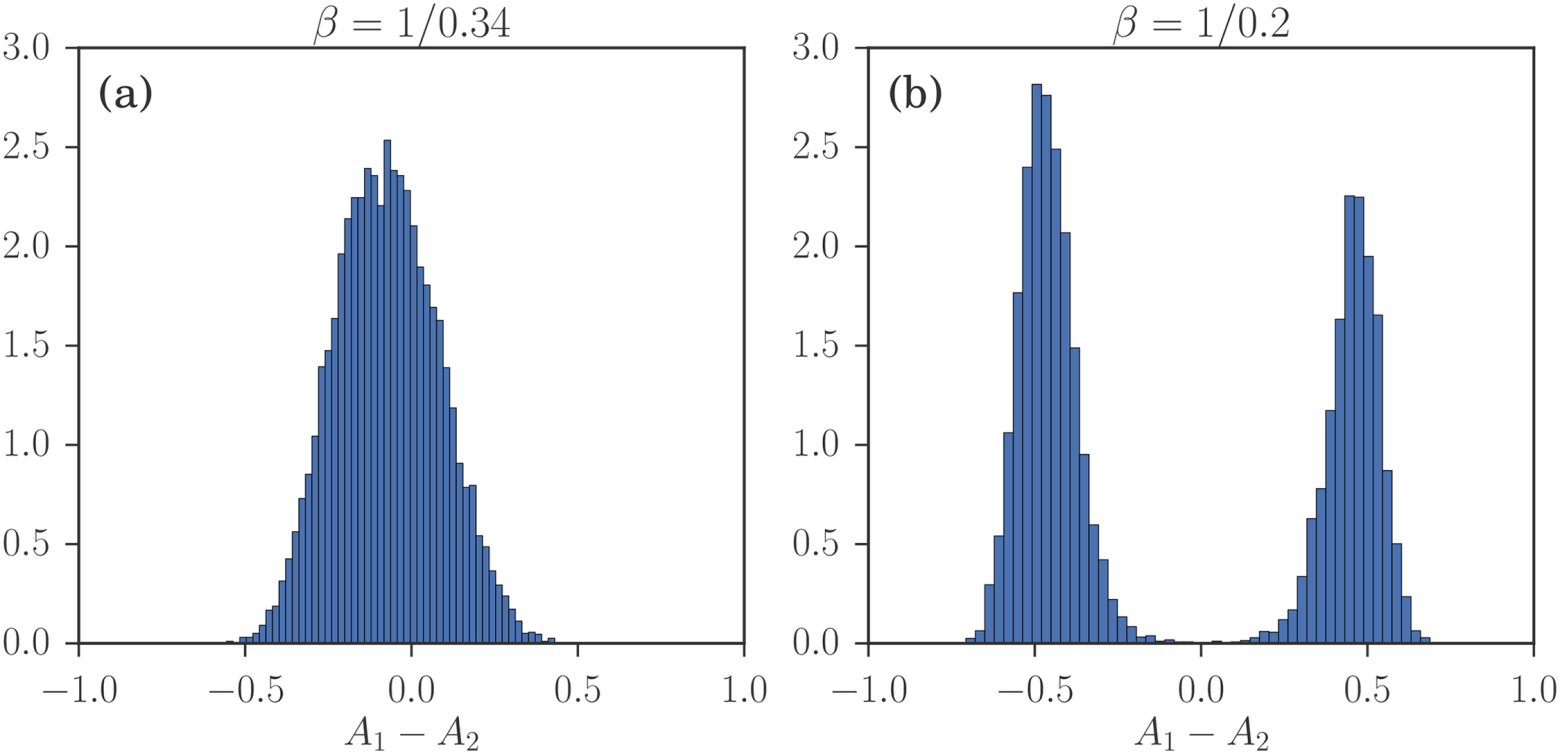
Results of a multi-agent simulation of the model of [6] after 5 ⋅ 10^4^ rounds of trading among 2 ⋅ 10^4^ agents. Parameters for the two markets are *θ*_1_ = 1 − *θ*_2_ = 0.3, buying preferences for the two classes of agents are $p_{\text{b}}^{\left(1\right)}=1-p_{\text{b}}^{\left(2\right)}=0.2$, forgetting rate *r* = 0.01 and *α* = 1 (no fictitious play). Shown is the distribution of attraction differences *A*_1_ − *A*_2_ across the first group of agents. This is unimodal for intensity of choice *β* below the SEHP threshold as in (a), but becomes bimodal for larger *β*: the system shows spontaneous emergence of preferences heterogeneity.

In the following we focus largely on a symmetric setup [6], explained in more detail when we classify Nash equilibria in the results section. There are two classes of agents in this scenario but their distributions of attractions are related by swapping *A*_1_ and *A*_2_ so it is enough to focus on one class. Numerical simulation and theoretical analysis of our reinforcement learning model, for *α* = 1, then show that when the intensity of choice *β* is above a threshold *β*_*c*_ the distribution of the traders’ attractions can become bi-modal [6]. By way of orientation, example simulation results for *β* both below and above the spontaneous emergence of heterogeneous preference (SEHP) threshold are shown in Fig 1.

### Incomplete versus complete information

One possible cause of heterogeneity in agents’ preferences that has been identified in previous studies is incomplete or imperfect information [24]. An obvious question is whether this explains the observation of spontaneous emergence of preferences heterogeneity in the double auction market model described above. Indeed, the agents in this model do have incomplete information about the markets they are trading in: they only receive the stochastic payoffs but do not have access to global information such as the number of buyers and sellers at each market, which they would need in order to estimate their average payoff. As a consequence, traders face the exploration/exploitation dilemma that is common in reinforcement learning [25]. They need to *explore* the whole strategy space (both high and low payoff strategies) to have accurate payoff estimates for their strategies, while at the same time *exploiting* the most profitable strategy by playing it frequently. In the model we consider the trade-off between exploration and exploitation is set by the intensity of choice *β* [26], with higher values favoring exploitation by making agents choose predominantly the market with the larger attraction.

To address the question of whether spontaneous emergence of preferences heterogeneity is possible also with *perfect* information, we develop in the next section an appropriate game theoretical version of the double auction model discussed above. Once we have determined the Nash equilibria of this game, we will come back to a comparison with the steady state of our reinforcement learning dynamics, to see how this resolves an indeterminacy in the Nash equilibria.

## Results and discussion: Mean field Nash equilibria

We now rephrase the double auction market choice model in game theoretical language. This will allow us to determine and classify its Nash equilibria in the mean field limit of an infinite number of players. Our aim will be to determine whether in this *perfect information* context there are still signatures of the spontaneous emergence of the phenomenon of preference heterogeneity previously found for our reinforcement learning approach with imperfect information. We will then see that, in the appropriate limit, the steady states of the reinforcement learning are consistent with the Nash equilibria of the model described in this section.

### Game theoretical framework

#### Setting

We consider a population of *N* traders called players (to be consistent with standard terminology in game theory). Those players are divided into two classes *c* ∈ {1, 2}, of the same size. Each player has fixed buy/sell preferences described by the probability to buy, $p_{\text{b}}^{\left(c\right)}$, which depends on his/her class. Each trading round is a round of the game, where each player chooses one of two actions, viz. “send an order to market one” and “send an order to market two”; we label these by *m* ∈ {1, 2}. A *pure strategy* is one where a player always chooses the same action. A *mixed strategy* is one where the player chooses action *m* = 1 with probability *p* ∈ [0, 1] and *m* = 2 otherwise. This formalism can be linked to our reinforcement learning model as described in the model section: there the traders learn which mixed strategy to play, mapping the learned attractions (*A*_1_, *A*_2_) to the probability *p* using the softmax function *σ*_*β*_(⋅) defined in Eq (3).

#### Average payoff in a large game

To determine the Nash equilibria, we need to determine the average payoff of a player for a given strategy *p*, given the (fixed) strategies of all other players. While this calculation would be complicated for finite *N*, it simplifies in the limit *N* → ∞ that we consider from now on. Firstly, the trading price at each market becomes non-fluctuating as the average value of bids and asks submitted becomes equal respectively to *μ*_b_ and *μ*_a_, up to fluctuations that vanish as $O(1/\sqrt{N})$.

Secondly, the ratio of the number of buyers and sellers at each market *m*, which we denote *f*_*m*_, also becomes non-fluctuating. We can calculate these ratios from the strategy distribution *ϕ*^(*c*)^(*p*) within each class of players, where because of the large *N*-limit we can neglect the effect of the strategy chosen by of any single player to obtain

$$\begin{array}{c} f_{1}\left(\phi^{\left(1\right)},\phi^{\left(2\right)}\right)=\frac{p_{\text{b}}^{\left(1\right)}{\bar{p}}^{\left(1\right)}+p_{\text{b}}^{\left(2\right)}{\bar{p}}^{\left(2\right)}}{\left(1-p_{\text{b}}^{\left(1\right)}\right){\bar{p}}^{\left(1\right)}+\left(1-p_{\text{b}}^{\left(2\right)}\right){\bar{p}}^{\left(2\right)}} \end{array}$$ (4)

$$\begin{array}{c} f_{2}\left(\phi^{\left(1\right)},\phi^{\left(2\right)}\right)=\frac{p_{\text{b}}^{\left(1\right)}\left(1-{\bar{p}}^{\left(1\right)}\right)+p_{\text{b}}^{\left(2\right)}\left(1-{\bar{p}}^{\left(2\right)}\right)}{\left(1-p_{\text{b}}^{\left(1\right)}\right)\left(1-{\bar{p}}^{\left(1\right)}\right)+\left(1-p_{\text{b}}^{\left(2\right)}\right)\left(1-{\bar{p}}^{\left(2\right)}\right)} \end{array}$$ (5)

Here ${\bar{p}}^{\left(c\right)}=\int \text{d}p\;\phi^{\left(c\right)}\left(p\right)p$ is the average mixed strategy parameter *p* in class *c*. In the above formulas, $Np_{\text{b}}^{\left(1\right)}{\bar{p}}^{\left(1\right)}$ is the typical number of agents of class 1 choosing to buy and to send their buy order to market 1. The relative fluctuations of this number again vanish for *N* → ∞. The other terms in the expressions for the *f*_*m*_ have analogous interpretations, and the common factor of *N* cancels.

Based on the above considerations, it becomes a simple matter to calculate the average payoff $P_{\tau ,m}\left(f_{m}\right)$ of buying (*τ* = b) or selling (*τ* = a) in market *m*, depending on the market conditions as encoded by *f*_*m*_. Our game is therefore *aggregative* [27]: average payoffs are determined only by the *aggregate* quantities *f*_1_ and *f*_2_ that can be calculated from the strategy distributions *ϕ*^(*c*)^(*p*). Other games in this class include the Cournot oligopoly; in statistical physics language the aggregates would be called order parameters.

In our setup we need to average the payoff $P_{\tau ,m}\left(f_{m}\right)$ further over the probability of buying or selling, giving for a player of class *c* an average payoff for the action of “going to market *m*” of

$$\begin{array}{c} P_{m}^{\left(c\right)}\left(f_{m}\right)=p_{\text{b}}^{\left(c\right)}P_{\text{b},m}\left(f_{m}\right)+\left(1-p_{\text{b}}^{\left(c\right)}\right)P_{\text{a},m}\left(f_{m}\right) \end{array}$$ (6)

Finally, for a player using a mixed strategy, the resulting payoff $P^{\left(c\right)}\left(p,f_{1},f_{2}\right)$ is an average of the payoff at market 1 weighted by *p* and the payoff at market 2 weighted by 1 − *p*:

$$\begin{array}{c} P^{\left(c\right)}\left(p,f_{1},f_{2}\right)=pP_{1}^{\left(c\right)}\left(f_{1}\right)+\left(1-p\right)P_{2}^{\left(c\right)}\left(f_{2}\right) \end{array}$$ (7)

This quantity is the key input into the calculation of the Nash equilibria of our game.

#### Nash equilibria

We choose to use the following definition of a Nash equilibrium for our game in the limit of an infinite number of players [11]. This definition takes advantage of the fact that we exploited in the payoff calculation, namely that for *N* → ∞ the aggregate quantities *f*_1_ and *f*_2_ remain constant if a single player changes strategy; in other words, players do not have market impact and their payoff depends only on their own strategy and the *distribution* of the strategies in the population overall.

**Definition 1.** *Nash equilibrium: The strategy distributions* *ϕ*^(1)^ *and* *ϕ*^(2)^ *constitute a Nash equilibrium of the game if the two following conditions are verified*:

$$\begin{array}{c} \text{Support}\left(\phi^{\left(1\right)}\right)\subseteq {\text{argmax}}_{p}\left(P^{\left(1\right)}\left(p,f_{1}\left(\phi^{\left(1\right)},\phi^{\left(2\right)}\right),f_{2}\left(\phi^{\left(1\right)},\phi^{\left(2\right)}\right)\right)\right) \end{array}$$ (8)

$$\begin{array}{c} \text{Support}\left(\phi^{\left(2\right)}\right)\subseteq {\text{argmax}}_{p}\left(P^{\left(2\right)}\left(p,f_{1}\left(\phi^{\left(1\right)},\phi^{\left(2\right)}\right),f_{2}\left(\phi^{\left(1\right)},\phi^{\left(2\right)}\right)\right)\right) \end{array}$$ (9)

*Here the maximization of the payoff on the right hand side is performed over the variable* *p* *at constant* *ϕ*^(*c*)^; *i.e*. *each single player maximizes their payoff with the aggregate quantities fixed*.

In words, the definition means that any strategy that has nonzero probability of being played by a player from class *c* (*i.e*. in the support of *ϕ*^(*c*)^) must maximize the player’s payoff. We will now apply this definition to determine the different classes of Nash equilibria that exist in the double auction market choice game.

### Classification of Nash equilibria

#### Equal payoff constraints

We will classify Nash equilibria according to two characteristics. If all agents in a class play the same strategy $p={\bar{p}}^{\left(c\right)}$, the distribution *ϕ*^(*c*)^(*p*) is a delta-distribution $\delta (p-{\bar{p}}^{\left(c\right)})$ and we call the equilibrium *homogeneous* for that class, otherwise—when different players in the same class use different *p*— we refer to the equilibrium as *heterogeneous*. The second characteristic is the strategy type: if all agents in a class play the pure strategies *p* = 0 or *p* = 1 we call the equilibrium *pure*, otherwise *mixed*. Combining these two characteristics then divides equilibria for each class into four possible types.

To obtain a classification of the possible overall Nash equilibria, note that the function being maximized in Eqs (8) and (9), viz. $p\to P^{\left(c\right)}\left(p,f_{1}\left(\phi^{\left(c\right)},\phi^{\left(2\right)}\right),f_{2}\left(\phi^{\left(1\right)},\phi^{\left(2\right)}\right)\right)$ is *linear* in *p*. As a consequence, if it is not constant, it has a single maximum on one of the boundaries of the interval [0, 1] where it is defined. A glance at (7) shows that the payoff function is constant if and only if *ϕ*^(1)^ and *ϕ*^(2)^ are such that the payoffs at the two markets are equal:

$$\begin{array}{c} P_{1}^{\left(c\right)}\left(f_{1}\left(\phi^{\left(1\right)},\phi^{\left(2\right)}\right)\right)=P_{2}^{\left(c\right)}\left(f_{2}\left(\phi^{\left(1\right)},\phi^{\left(2\right)}\right)\right) \end{array}$$ (10)

If (and only if) this *equal payoff condition* is satisfied, the strategy distribution *ϕ*^(*c*)^(*p*) can be nonzero for any *p* ∈ [0, 1]. This can be interpreted by saying that, if in a class there are players that go the first and the second market, the only way for none of them to have an incentive to move to another market is for the payoff at the two markets to be the same.

If the equal payoff condition is not met for a class, we have to have either

$$\begin{array}{c} P_{1}^{\left(c\right)}\left(f_{1}\left(\phi^{\left(1\right)},\phi^{\left(2\right)}\right)\right)>P_{2}^{\left(c\right)}\left(f_{2}\left(\phi^{\left(1\right)},\phi^{\left(2\right)}\right)\right),\;\phi^{\left(c\right)}\left(p\right)=\delta \left(p-1\right),\;{\bar{p}}^{\left(c\right)}=1 \end{array}$$ (11)

or

$$\begin{array}{c} P_{1}^{\left(c\right)}\left(f_{1}\left(\phi^{\left(1\right)},\phi^{\left(2\right)}\right)\right)<P_{2}^{\left(c\right)}\left(f_{2}\left(\phi^{\left(1\right)},\phi^{\left(2\right)}\right)\right),\;\phi^{\left(c\right)}\left(p\right)=\delta \left(p\right),\;{\bar{p}}^{\left(c\right)}=0 \end{array}$$ (12)

In both cases the strategy distribution is homogeneous pure, and the entire class of agents goes to the market with the higher payoff.

#### Types of Nash equilibria

**Fig 2:**
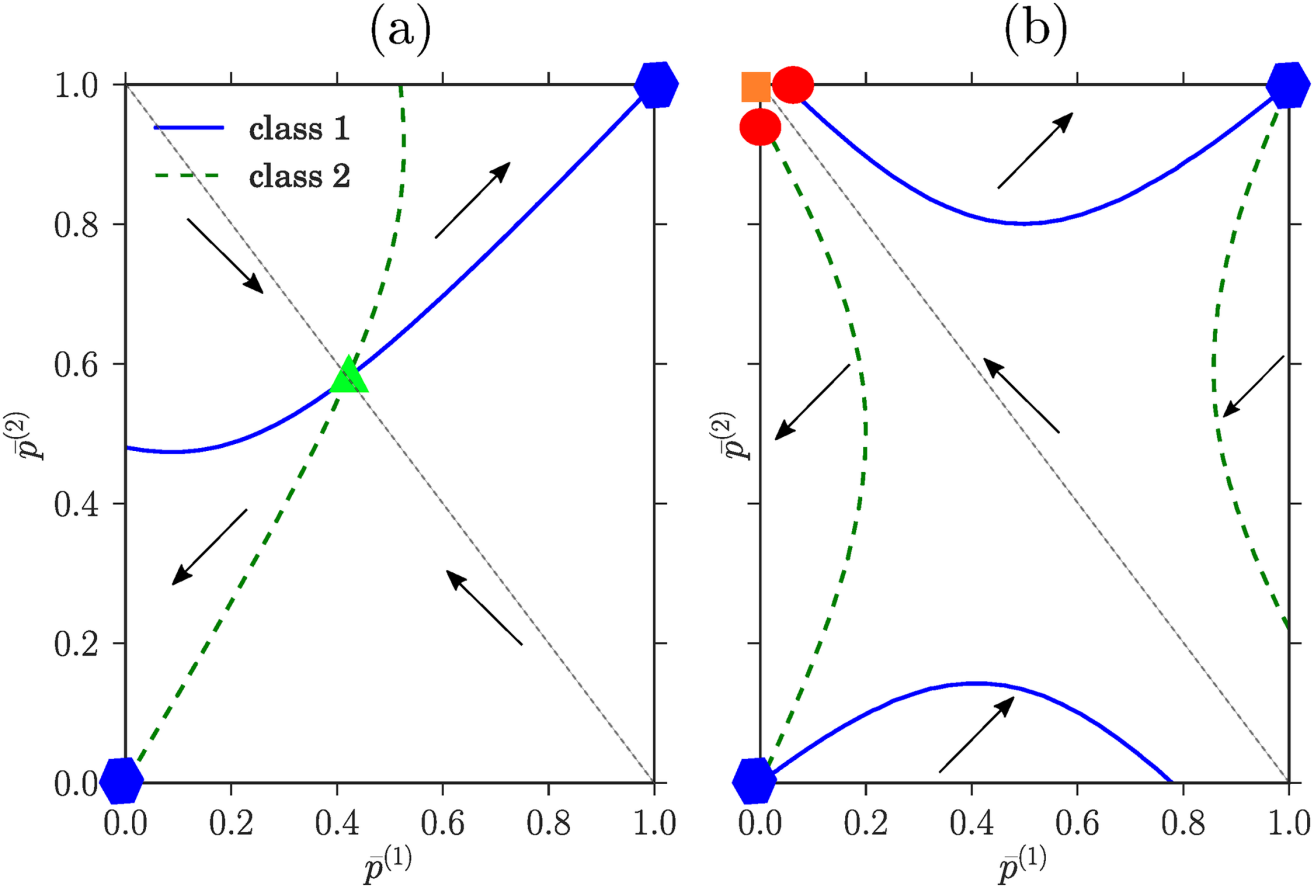
Values of ${\bar{p}}^{\left(1\right)},{\bar{p}}^{\left(2\right)}$ for which the equal payoff constraints are verified for class *c* = 1 (blue, solid) and class *c* = 2 (green, dashed). The arrows point to (*s*^(1)^, *s*^(2)^) where *s*^(*c*)^ ∈ {0, 1} indicates the profit-maximizing strategy of traders from class *c*, in each distinct area of the plane. In panel (a) where *θ*_1_ = 1 − *θ*_2_ = 0.3, $p_{\text{b}}^{\left(1\right)}=1-p_{\text{b}}^{\left(2\right)}=0.2$, there exists a heterogeneous equilibrium (green triangle), located at the intersection of the two equal payoff curves. In panel (b), *θ*_1_ = 1 − *θ*_2_ = 0.2, $p_{\text{b}}^{\left(1\right)}=1-p_{\text{b}}^{\left(2\right)}=0.45$, and the equal payoff curves do not cross. There is then no potentially heterogeneous Nash equilibrium, but the direction of the arrows shows that a homogeneous pure equilibrium (orange square) with the two classes going to different markets exists. There are also two partially heterogeneous Nash equilibria (red circles, see main text). In both (a) and (b) there exist homogeneous pure Nash equilibria where the whole population trades at the same market (blue hexagons). The dotted line indicates the location of the symmetric equilibria that we mostly focus on.

We can now proceed to find the possible types of overall Nash equilibria for our game. Because *f*_1_ and *f*_2_ are fixed once ${\bar{p}}^{\left(1\right)}$ and ${\bar{p}}^{\left(2\right)}$ are known, the equal payoff condition for each class defines a line of points in the $\left({\bar{p}}^{\left(1\right)},{\bar{p}}^{\left(2\right)}\right)$ plane. This line can consist of several distinct pieces as shown in the examples in Fig 2, where equal payoff lines are plotted for both class *c* = 1 (full lines) and *c* = 2 (dashed lines).

The discussion above can now be summarized in graphical terms as follows: a point in the $\left({\bar{p}}^{\left(1\right)},{\bar{p}}^{\left(2\right)}\right)$-plane is a Nash equilibrium if for each class the point is either on the equal payoff line, or on the boundary (specified by ${\bar{p}}^{\left(c\right)}=1$ or = 0) corresponding to the market where the class has the higher payoff. Combining these options for the two classes, the first and for our purposes most interesting type of Nash equilibrium that results is a point at an intersection of two equal payoff lines, away from the boundaries. We call such a point a *potentially heterogeneous* Nash equilibrium. Here both ${\bar{p}}^{\left(1\right)}$ and ${\bar{p}}^{\left(2\right)}$ are strictly between 0 and 1. The strategy distributions can then be either

- homogeneous mixed, with $\phi^{\left(c\right)}=\delta \left(p-{\bar{p}}^{\left(c\right)}\right)$, or
- heterogeneous pure, with $\phi^{\left(c\right)}=\left(1-{\bar{p}}^{\left(c\right)}\right)\delta \left(p\right)+{\bar{p}}^{\left(c\right)}\delta \left(p-1\right)$, or
- heterogeneous mixed otherwise.

**Fig 3:**
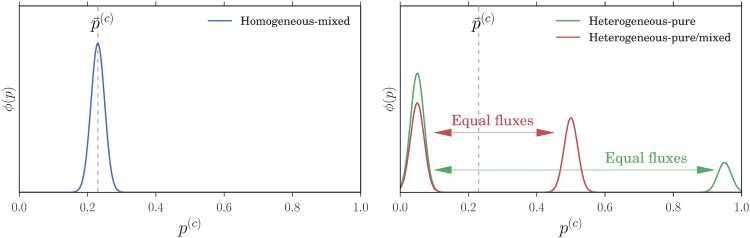
Three different types of strategy distribution *ϕ*(*p*) that all have the same mean $\bar{p}$ (dashed line): Homogeneous mixed distribution (left panel), heterogeneous mixed (red curve, right panel) heterogeneous pure (green curve, right panel). Peaks in the distribution are shown broadened as they would be in our reinforcement learning model at finite decision strength *β*; as Nash equilibria they would become sharp (delta-distributions). The right panel illustrates that, when a strategy distribution has two distinct peaks, it can represent a steady state of the learning dynamics only when the fluxes of agents moving from one peak to the other balance in the two directions (see the Methods section).

These three different cases are illustrated schematically in Fig 3. The homogeneous mixed case can be viewed as the Nash equilibrium analogue of the unimodal distribution in the stochastic simulations shown in Fig 3; in the heterogeneous mixed case the strategy distribution is arbitrary except for its fixed mean ${\bar{p}}^{\left(c\right)}$. The fact that the Nash equilibrium conditions here allow both homogeneous and heterogeneous strategy distributions motivates our use of the term “potentially heterogeneous”. It also shows that one needs dynamical information to say more about the strategy distribution shapes, as explored in detail in the results section.

A second type of Nash equilibrium results when the equal payoff condition is obeyed for only one class while the other class is at a boundary. We then speak of a *partially potentially heterogeneous* Nash equilibrium, because one class of players has a homogeneous pure strategy distribution while the other strategy distribution is of one of the three types listed in the bullet points above.

Finally, Nash equilibria unconstrained by either of the equal payoff conditions must be in on of the four corners of the square $\left({\bar{p}}^{\left(1\right)},{\bar{p}}^{\left(2\right)}\right)\in {\left[0,1\right]}^{2}$; we call them *homogeneous pure* equilibria as the strategy distributions for both classes are then of this type. These equilibria can be further subdivided depending on whether both classes go to the same market or not. The former type always exists as if one of the traders tries to trade in the empty market s/he will earn a payoff of 0 which is smaller than the payoff s/he could earn in the non-empty market. In the latter type, each market is used only by traders of one class, who trade with each other there.

Plots in the $\left({\bar{p}}^{\left(1\right)},{\bar{p}}^{\left(2\right)}\right)$-plane as shown in Fig 2 are a convenient graphical tool to assess the existence of potentially heterogeneous, potentially partially heterogeneous and homogeneous pure Nash equilibria. Potentially heterogeneous equilibria are found directly as interior crossing points of the equal payoff curves for the two classes. A partially heterogeneous Nash equilibrium corresponds to a point (see Fig 2(b)) that is located at the intersection of the equal payoff curve of class 1 (resp. 2) and a horizontal (resp. vertical) boundary. This criterion identifies a list of (usually four) candidate equilibria. To have an actual equilibrium the payoffs of the markets for the homogeneous pure class need to have the correct order, e.g. for a candidate point located on the axis ${\bar{p}}^{\left(2\right)}=1$, the payoff at market 1 has to be higher for class 2 players than the payoff at market 2. By drawing arrows indicating payoff ordering as explained in the caption of Fig 2, this can be summarized by saying that the arrows must point *towards* the boundary that a candidate point for a potentially partially heterogeneous Nash equilibrium lies on. In Fig 2, this leaves two equilibria of this type as marked by the red circles.

Finally, for a heterogeneous pure Nash equilibrium where the two classes of players choose different markets, the two candidate points are the top left or bottom right corner. These are again Nash equilibria provided they have the correct ordering of payoffs, which requires that the arrows drawn in the figure point towards this corner. In Fig 2(b) this is the case for the top left corner (orange square).

**Fig 4:**
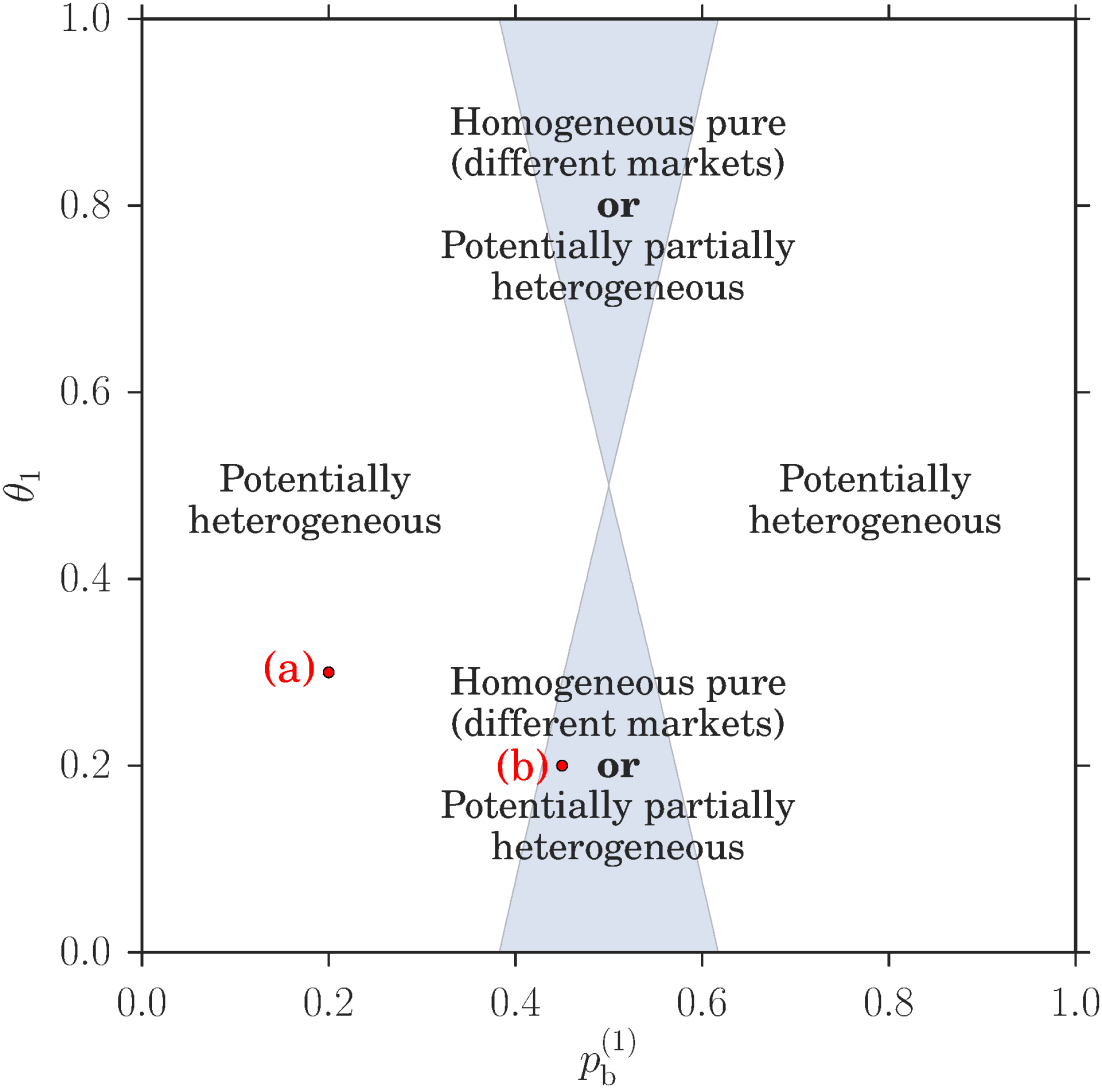
Phase diagram for existence of different types of Nash equilibria for a system with symmetric price setting parameters *θ*_1_ = 1 − *θ*_2_ and buying preferences $p_{\text{b}}^{\left(1\right)}=1-p_{\text{b}}^{\left(2\right)}$. The types of equilibria in this plot are explained in the results section and a graphical method to check their existence is shown in Fig 2. The labels (a) and (b) correspond to the panels there. Note that the two homogeneous pure Nash equilibria where both classes of player trade at the same market are not shown as they exist everywhere.

We can now look at how the existence of the different types of Nash equilibria depends on the system parameters, which are the market biases *θ*_*m*_ and the buying preferences $p_{\text{b}}^{\left(c\right)}$. We follow Ref. [6] in focusing on a symmetric setup where the two markets have opposite biases in favour of buyers and sellers. As *θ* = 0.5 corresponds to the absence any bias, this means *θ*_1_ + *θ*_2_ = 1. Similarly we assume that the players fall into two symmetric groups with respect to their buying preferences, with those in class 1 preferring to buy ($p_{\text{b}}^{\left(1\right)}<0.5$) and the others having the opposite preference $p_{\text{b}}^{\left(2\right)}=1-p_{\text{b}}^{\left(1\right)}$. With these choices, we can show in Fig 4 the regions where the different types of Nash equilibria exist as a function of $p_{\text{b}}^{\left(1\right)}$ and *θ*_1_. It turns out that the two examples shown in Fig 2 cover the two generic cases: in addition to homogeneous pure Nash equilibria where both classes go to the same market, which always exist, one has either a potentially heterogeneous Nash equilibrium as in Fig 2(a), or a homogeneous pure equilibrium with the two classes at different markets and two potentially partially heterogeneous equilibria (Fig 2(b)). These two cases are mutually exclusive. An analytical expression for the boundary between the zones where they exist can also be obtained as detailed in the methods section.

Returning to the broader picture, the Nash equilibrium analysis of the double auction market choice game clearly shows that there is *potential* for spontaneous emergence of preferences heterogeneity: as illustrated in Fig 3, heterogeneous pure strategy distributions have two peaks that indicate players within a class separating into two distinct subpopulations playing opposite pure strategies. Heterogeneous mixed strategies can similarly have two or more peaks. This shows that the observaions of spontaneous emergence of preferences heterogeneity, also called segregation in a previous study of our reinforcement learning model [6] were not based on purely dynamical effects. We also find qualitatively similar trends, e.g. the equilibria where both classes of players can be segregated (potentially heterogeneous) are most prevalent in Fig 4 when the two markets are identical (*θ*_1_ = 0.5), showing that the spontaneous emergence of preferences heterogeneity is not a trivial consequence of differences between markets.

However, the Nash equilibrium conditions only identify the means of the strategy distributions *ϕ*^(1)^ and *ϕ*^(2)^. As we saw, this means for a potentially heterogeneous (or potentially partially heterogeneous) equilibrium that we cannot decide whether the underlying strategy distribution is homogeneous (mixed) or heterogeneous, nor do we know whether a heterogeneous mixed strategy distribution would actually have two distinct peaks as required for the concept of segregation to make sense. We therefore study next under what conditions our reinforcement learning *dynamics* as defined in the model section reaches as its steady state a Nash equilibrium of our system. Once this connection is established, we ask which particular Nash equilibria are selected as possible steady states of our reinforcement learning dynamics. Put differently, does the learning dynamics break the indeterminacy of the Nash equilibrium conditions?

## Results and discussion: Reinforcement learning in double auction markets

In this section, we study the steady states of our reinforcement learning dynamics defined in the model section in a game with a large number of players. We are interested in particular when different types of steady state strategy distributions, as sketched in Fig 3, can occur.

**Fig 5:**
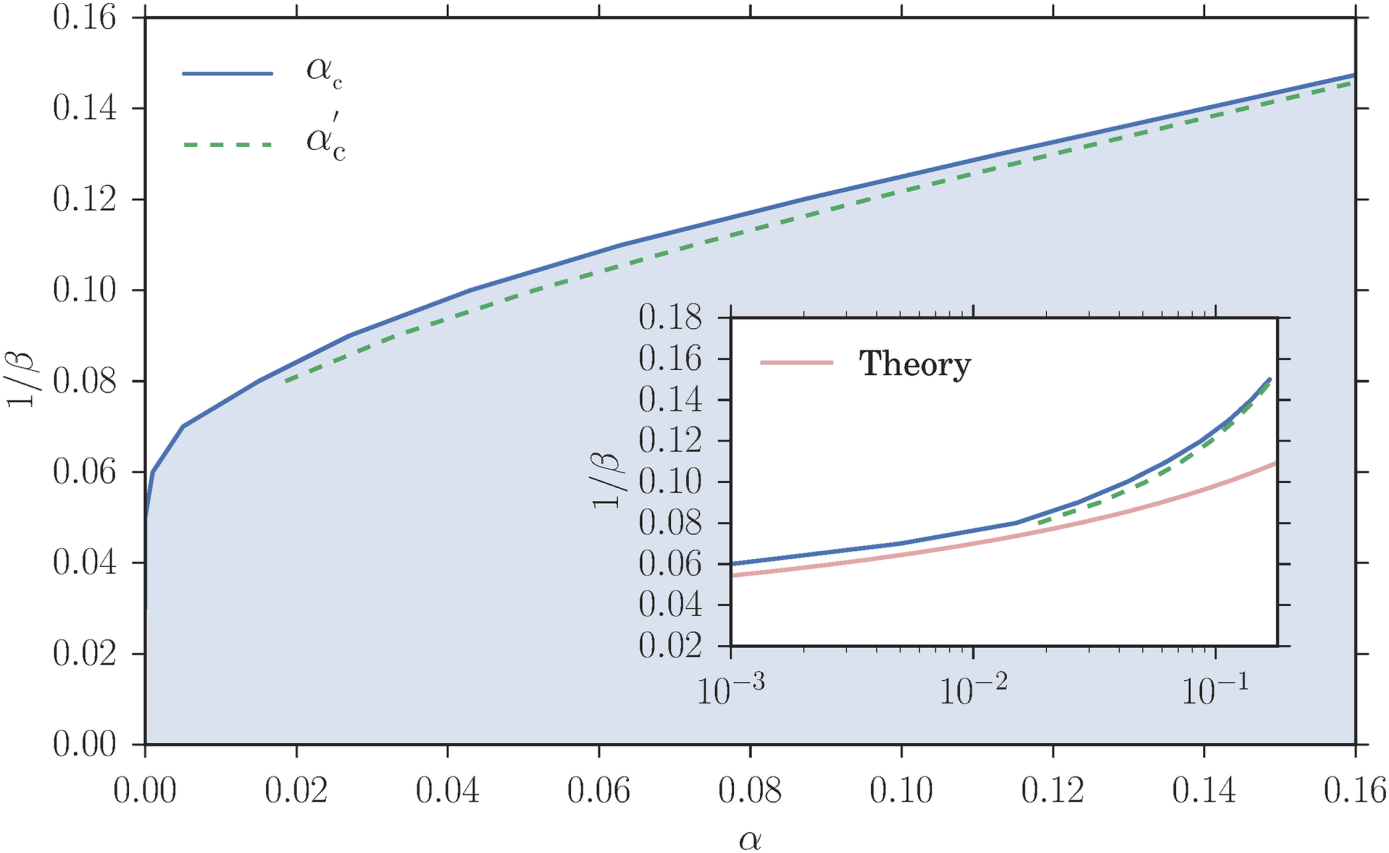
Phase diagram of our reinforcement learning model. The blue zone shows the region of the (*α*, 1/*β*)-plane where the steady state strategy distribution of each of the two classes of agents is heterogeneous. Elsewhere, including in particular on the line *α* = 0, the strategy distribution is homogeneous. The blue line shows the threshold *α*_*c*_ where the distribution switches from homogeneous to heterogeneous mixed. As *α* is increased further beyond a threshold $\alpha_{c}^{\prime }$ (dashed green line), the strategy distribution becomes heterogeneous pure. The market and trader parameters for this diagram are *θ*_1_ = 1 − *θ*_2_ = 0.3 and $p_{\text{b}}^{\left(1\right)}=1-p_{\text{b}}^{\left(2\right)}=0.2$. Inset: Threshold curves plotted with a logarithmic *α*-axis. The red line shows the exponential dependence of the characteristic values of *α* on *β* (with an arbitrary prefactor) that is expected from the theoretical considerations we describe in the methods section.

We argue in the results section that one expects the steady state of our reinforcement learning dynamics to approach a Nash equilibrium of the we described previously in the joint limit where the fictitious play coefficient *α* → 0, the intensity of choice *β* → ∞ and the inverse memory length *r* → 0. In principle our task is thus to find the steady state of our reinforcement learning and then to take this joint limit. It turns out, however, that this is far from trivial. The reason is shown by the phase diagram in Fig 5, where the limit *r* → 0 has already been taken. What is notable is that there are different regions in the phase diagram where the steady state strategy distributions are homogeneous and heterogeneous, respectively. The Nash equilibrium limit point (*α*, 1/*β*) = (0, 0) can be approached along paths within either of these regions, which means there will be several possible limiting strategy distributions of our reinforcement learning dynamics, and it is these that we will want to identify. Note that we focus generally on system parameters where potentially heterogeneous Nash equilibria exist (see Fig 4), for which the phase diagram of our reinforcement learning model has the generic structure of Fig 5.

We introduce in the methods section, the Kramers-Moyal expansion for our reinforcement learning dynamics on which the rest of the analysis is based. In particular, we study homogeneous and heterogeneous distributions of preferences, and analyse how they approach Nash equilibria in the relevant limit. The large deviation methods we deploy for the heterogeneous case are described separately in the methods section as well.

As before we choose to concentrate on settings with symmetric market biases and buy/sell preferences, and within those on steady states of our learning dynamics that also have symmetric aggregates ${\bar{p}}^{\left(1\right)}=1-{\bar{p}}^{\left(2\right)}$. This captures the dominant steady states, simplifies the numerical analysis (see the Methods section) and also makes it easier to illustrate the concepts. In the graphical representation of Fig 2, the steady states we are considering lie on the diagonal from top left to bottom right (dotted line).

### Nash equilibria as limits of reinforcement learning

In the game theoretical study above, we considered a large game (*N* → ∞). The Nash equilibria we studied assume implicitly (i) that each player is able to evaluate his expected payoff (*full information assumption*), (ii) that this evaluation averages appropriately over all stochastic effects (*no fluctuation assumptions*) and (iii) that the players always choose the action with the highest payoff (*best response assumption*). One therefore expects a learning dynamics that verifies these same assumptions to converge to one of the Nash equilibria we characterized in the previous section.

We now consider when the above assumptions hold for our reinforcement learning dynamics. If we want the players’ attractions to be accurate estimates of the payoffs for the corresponding action (assumption (i)) we require *α* → 0 to ensure that the attractions to actions that are not played do not decrease over time. To average over payoff fluctuations (assumption (ii)) we further need to work in the large memory limit *r* → 0. To see this, note that in each training round the players’ attractions are modified only by an amount of order *r*. For small *r*, attractions therefore change substantially after ∼1/*r* training rounds. This means the players effectively average the payoffs over many trading rounds that take place while their attractions and hence their strategies remain fixed, and in the limit obtain the correct expected payoffs [28]. Finally, a large intensity of choice (*β* → ∞) ensures that players best respond to their attractions, so that our reinforcement learning model in that limit also verifies assumption (iii).

### Kramers-Moyal expansion for *r* → 0

Of the three limits identified above we take first the large memory limit *r* → 0. In this limit—and the large system limit *N* → ∞, which we always assume—the dynamics of our reinforcement learning model can be described by a (nonlinear) Fokker-Planck equation [6]. This is derived by a Kramers-Moyal expansion truncated at the second order; we defer the details to the methods section. Denoting by $P(A^{\left(c\right)},t)$ the distribution of attractions of traders from class *c*, where $A^{\left(c\right)}=\left(A_{1}^{\left(c\right)},A_{2}^{\left(c\right)}\right)$ is a vector gathering the attractions towards market 1 and 2, the Fokker-Planck equation describing the time evolution of this distribution is

$$\begin{array}{ccc} \partial_{t}P\left(A^{\left(c\right)},t\right) & = & -\sum_{m=1}^{2}\partial_{A_{m}^{\left(c\right)}}\left[\mu_{m}^{\left(c\right)}\left(A^{\left(c\right)},{\bar{p}}^{\left(1\right)},{\bar{p}}^{\left(2\right)}\right)P\left(A^{\left(c\right)},t\right)\right]\\ & & +\frac{r}{2}\sum_{m,m^{\prime }=1}^{2}\partial_{A_{m}^{\left(c\right)}}\partial_{A_{m^{\prime }}^{\left(c\right)}}\left[\Sigma_{mm^{\prime }}^{\left(c\right)}\left(A^{\left(c\right)},{\bar{p}}^{\left(1\right)},{\bar{p}}^{\left(2\right)}\right)P\left(A^{\left(c\right)},t\right)\right] \end{array}$$ (13)

Here time *t* = *rn* is a rescaled version of the number of trading rounds *n*, while ${\bar{p}}^{\left(1\right)}$ and ${\bar{p}}^{\left(2\right)}$ are the average fractions of traders from class 1 (resp. class 2) choosing to go to the first market. These fractions are obtained simply by averaging the probability of choosing market 1 as defined in (3) over the relevant distribution of attractions:

$$\begin{array}{c} {\bar{p}}^{\left(c\right)}=\int \text{d}A^{\left(c\right)}\;P\left(A^{\left(c\right)},t\right)\sigma_{\beta }\left(A_{1}^{\left(c\right)}-A_{2}^{\left(c\right)}\right) \end{array}$$ (14)

Formally, ${\bar{p}}^{\left(1\right)}$ and ${\bar{p}}^{\left(2\right)}$ are therefore functionals of the probability distributions $P(A^{\left(c\right)},t)$ It is this dependence that makes the Fokker-Planck equation nonlinear, and couples the dynamics of the attraction distributions in class 1 and 2.

At fixed values of ${\bar{p}}^{\left(1\right)}$ and ${\bar{p}}^{\left(2\right)}$, the Fokker-Planck equation (13) describes for each class the Langevin dynamics of the attraction vector **A**^(*c*)^ of a *single agent*, with deterministic drift vector $\mu_{m}^{\left(c\right)}$ and (multiplicative) white noise with covariance matrix $r\Sigma_{mm^{\prime }}^{\left(c\right)}$. The form of the drift follows directly from the original reinforcement learning dynamics (2) (see the Methods section)

$$\begin{array}{c} \mu_{1}^{\left(c\right)}\left(A^{\left(c\right)},{\bar{p}}^{\left(1\right)},{\bar{p}}^{\left(2\right)}\right)=\left[P_{1}^{\left(c\right)}\left(f_{1}\left({\bar{p}}^{\left(1\right)},{\bar{p}}^{\left(2\right)}\right)\right)-A_{1}^{\left(c\right)}\right]\sigma_{\beta }\left(A_{1}^{\left(c\right)}-A_{2}^{\left(c\right)}\right)-\alpha A_{1}^{\left(c\right)}\left[1-\sigma_{\beta }\left(A_{1}^{\left(c\right)}-A_{2}^{\left(c\right)}\right)\right] \end{array}$$ (15)

The first term describes the change in the attraction to market 1 (in square brackets), weighted with the probability of the agent choosing that market. The second term corresponds to the opposite case where the agent chooses market 2.

The Fokker-Planck equation (13) is of course impossible to solve in closed form in general. A special case is the limit *r* → 0, assuming the population is initially homogeneous, *i.e*. a delta-distribution. Homogeneity is then maintained over time for *r* = 0, where the dynamics is deterministic, and Eq (13) gives for the time evolution of the locations of the peaks of the attraction distributions the equations

$$\begin{array}{c} \partial_{t}A_{m}^{\left(c\right)}=\mu_{m}^{\left(c\right)}\left(A^{\left(c\right)},{\bar{p}}^{\left(1\right)},{\bar{p}}^{\left(2\right)}\right) \end{array}$$ (16)

Together with

$$\begin{array}{c} {\bar{p}}^{\left(c\right)}\left(t\right)=\sigma_{\beta }\left(A_{1}^{\left(c\right)}\left(t\right)-A_{2}^{\left(c\right)}\left(t\right)\right) \end{array}$$ (17)

one then has a system of nonlinear differential equations that is straightforward to solve numerically. We call this the *homogeneous populations dynamics*, where the population changes over time but remains homogeneous.

For nonzero *r*, analysing the Fokker-Planck equation becomes more difficult because the attraction distributions broadens and can indeed develop multiple peaks. As we are primarily interested in long-time steady states, we focus on this somewhat simpler case. The task at hand here is a self-consistency problem: find a set of aggregates ${\bar{p}}^{\left(1\right)}$, ${\bar{p}}^{\left(2\right)}$ for which the steady state solution of the Fokker-Planck equation, when inserted into (14), gives back the original aggregates. If we call ${\tilde{p}}^{\left(c\right)}\left({\bar{p}}^{\left(1\right)},{\bar{p}}^{\left(2\right)}\right)$ the aggregates calculated from the steady state solution, the self-consistency equations are simply ${\tilde{p}}^{\left(c\right)}\left({\bar{p}}^{\left(1\right)},{\bar{p}}^{\left(2\right)}\right)={\bar{p}}^{\left(c\right)}$.

### Steady state of the Fokker-Planck equation

The remaining challenge is now to determine, for small *r*, the steady state solution of the Fokker-Planck equation for given aggregates ${\bar{p}}^{\left(1\right)},{\bar{p}}^{\left(2\right)}$. As explained above, we can think of this as the steady state distribution for the dynamics of a single agent, given a fixed state of the population. In the limit *r* → 0 this dynamics is almost deterministic so that the agent will spend almost all of her/his time near the stable fixed points of the drift $\mu_{m}^{\left(c\right)}$. Accordingly, $P(A^{\left(c\right)})$ will be peaked near these points, with the peak width being of the order of the standard deviation of the Langevin noise, *i.e*. $O(\sqrt{r})$.

**Fig 6:**
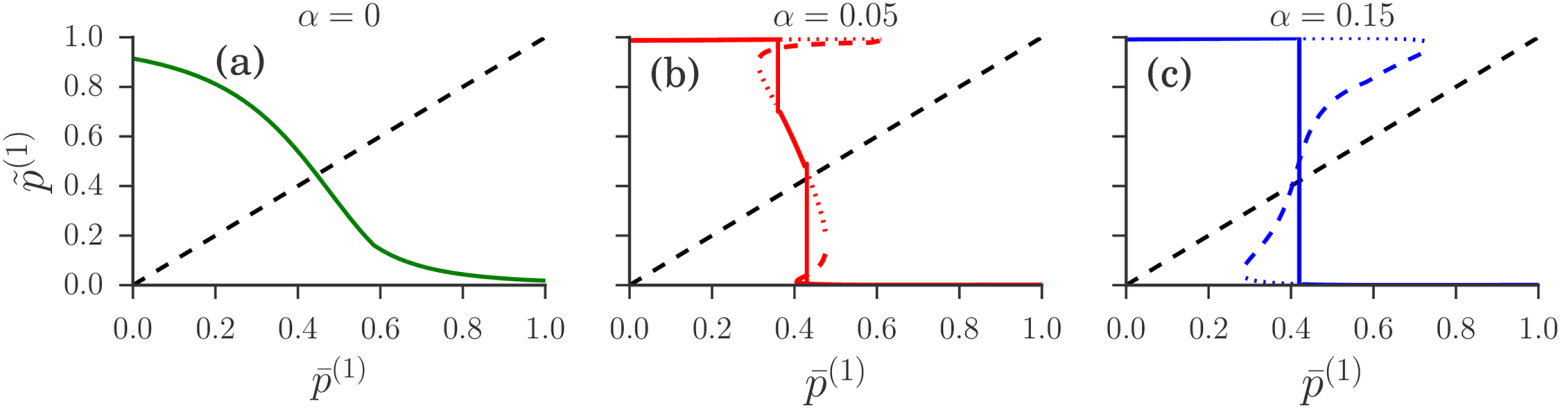
New aggregate ${\tilde{p}}^{\left(1\right)}$ calculated from steady state of single agent dynamics at “old” aggregate value ${\bar{p}}^{\left(1\right)}$ (for *r* → 0). Steady states are peaked around stable fixed points (solid/dotted), which are connected by unstable fixed points (dashed). In (a) only one such peak exists for any ${\bar{p}}^{\left(1\right)}$. The physical steady state is found from the self-consistency requirement ${\tilde{p}}^{\left(1\right)}={\bar{p}}^{\left(1\right)}$ (dot-dashed line). In (b, c) there are steady states with up to three peaks, but generically all but one have a weight exponentially suppressed in 1/*r* so that ${\tilde{p}}^{\left(1\right)}\left({\bar{p}}^{\left(1\right)}\right)$ (solid line) follows the curve for a single fixed point. At specific aggregate values the dominant peak switches and two peaks can coexist (vertical solid lines). In (b) there are two such transitions; in (c) the middle fixed point from (b) has disappeared and there is only one transition, between branches of ${\tilde{p}}^{\left(1\right)}$ that are close to 0 and 1. In (b, c) the intersection with the diagonal is *at* a switch, giving a heterogeneous steady state with two peaks of comparable weight. Market and trader parameters for this figure are as in Fig 5; intensity of choice *β* = 1/0.1.

For aggregate values where there is only one stable single agent fixed point, $P(A^{\left(c\right)})$ becomes a delta-distribution centred at that point for *r* → 0, so we have a steady state with a homogeneous distribution of attractions and hence strategies. The self-consistency condition for such a steady state is then simply the stationarity condition for the homogeneous population dynamics (16) together with (17). The graphical solution of this condition is illustrated in Fig 6(a).

When there are multiple stable single agent fixed points, $P(A^{\left(c\right)})$ for *r* → 0 will become a sum of delta-distributions at these points. The remaining task is then to find the *weight* of each of these peaks. We explain how to use *large deviation* methods for this purpose in the methods section. The idea is that the peak weights are determined by the balance of fluxes of agents transitioning from one peak to another. For small *r*, the dominant *r*-dependence of these fluxes comes from exponential factors of the form exp(-S/r). Fluxes can then balance for *r* → 0 only when the “action” S, which represents an effective activation barrier, is the same for the transition from one peak to the other as for the reverse transition. This condition, which is represented schematically in Fig 3, allows one to determine the aggregate values where multiple peaks can coexist in P(A). At these aggregate values the steady state solution switches between two single peaked solutions. This switch happens within an aggregate value range of *O*(*r*) that vanishes as *r* → 0, giving vertical sections in the plot of ${\tilde{p}}^{\left(c\right)}$ versus ${\bar{p}}^{\left(c\right)}$ as shown in Fig 6(b). If the intersection with the diagonal ${\tilde{p}}^{\left(c\right)}={\bar{p}}^{\left(c\right)}$ occurs in one of these vertical sections, as in the example in Fig 6(b), the actual peak weights can be determined indirectly from the fact that the appropriate weighted combination of the ${\tilde{p}}^{\left(c\right)}$ from the single peaks must give ${\bar{p}}^{\left(c\right)}$. Note that one can show generally (see the Methods section) that in each agent class there can be at most three stable fixed points, so that each $P(A^{\left(c\right)})$ can have at most three peaks. By choosing an appropriate aggregate value, at most two of these peaks can be made to have finite weight for *r* → 0. Obtaining three peaks with finite weight requires one to tune *α* to $\alpha_{c}^{\prime }$ at given *β*, giving the dashed green phase boundary in Fig 5. Intuitively, at $\alpha_{c}^{\prime }$ the two transitions in Fig 6(b) have moved horizontally so that they occur at the same aggregate value.

We will next study the homogeneous steady states of reinforcement learning dynamics. Given the structure of the phase diagram that we anticipated in Fig 5, the easiest way to ensure that steady states are homogeneous in the Nash equilibrium limit is to take *α* = 0.

### Homogeneous attraction distributions

#### Kramers-Moyal expansion for *α* = 0

We saw above that the dynamics of a homogeneous distributions of agents within each class is described, for *r* → 0 by (16 and 17). In steady state the right-hand side of (16) needs to vanish, hence using *α* = 0 in (15) and its analogue for *m* = 2 one has

$$\begin{array}{c} 0=\left[P_{1}^{\left(c\right)}\left(f_{1}\left({\bar{p}}^{\left(1\right)},{\bar{p}}^{\left(2\right)}\right)\right)-A_{1}^{\left(c\right)}\right]\sigma_{\beta }\left(A_{1}^{\left(c\right)}-A_{2}^{\left(c\right)}\right) \end{array}$$ (18)

$$\begin{array}{c} 0=\left[P_{2}^{\left(c\right)}\left(f_{2}\left({\bar{p}}^{\left(1\right)},{\bar{p}}^{\left(2\right)}\right)\right)-A_{2}^{\left(c\right)}\right]\sigma_{\beta }\left(A_{2}^{\left(c\right)}-A_{1}^{\left(c\right)}\right) \end{array}$$ (19)

Here the aggregates on which *f*_1_ and *f*_2_ depend are given by ${\bar{p}}^{\left(c\right)}=\sigma_{\beta }\left(A_{1}^{\left(c\right)}-A_{2}^{\left(c\right)}\right)$. In (19), $\sigma_{\beta }\left(A_{1}^{\left(c\right)}-A_{2}^{\left(c\right)}\right)$ cannot vanish at any finite *β*, so the condition for a homogeneous state is simply

$$\begin{array}{c} P_{m}^{\left(c\right)}\left(f_{1}\left({\bar{p}}^{\left(1\right)},{\bar{p}}^{\left(2\right)}\right)\right)-A_{m}^{\left(c\right)}=0 \end{array}$$ (20)

which needs to be verified for each market *m* and each class *c*. This means that for each player, in the steady state of the reinforcement learning dynamics, the respective attraction to each market equals the expected payoff there. The aggregates calculated from the steady state are therefore

$$\begin{array}{c} {\tilde{p}}^{\left(c\right)}\left({\bar{p}}^{\left(1\right)},{\bar{p}}^{\left(2\right)}\right)=\sigma_{\beta }\left(P_{1}^{\left(c\right)}\left(f_{1}\left({\bar{p}}^{\left(1\right)},{\bar{p}}^{\left(2\right)}\right)\right)-P_{2}^{\left(c\right)}\left(f_{2}\left({\bar{p}}^{\left(1\right)},{\bar{p}}^{\left(2\right)}\right)\right)\right) \end{array}$$ (21)

We now need to solve the self-consistency condition ${\tilde{p}}^{\left(c\right)}={\bar{p}}^{\left(c\right)}$ as explained in the results section. This can be visualized most easily if we focus on symmetric situations where ${\bar{p}}^{\left(1\right)}=1-{\bar{p}}^{\left(2\right)}$: one just has to plot the curve $\sigma_{\beta }\left(P_{1}^{\left(1\right)}-P_{2}^{\left(1\right)}\right)$ vs ${\bar{p}}^{\left(1\right)}$ and intersect it with the diagonal, as shown in Fig 6(a).

To retrieve our reinforcement learning steady states corresponding to Nash equilibria, we need to consider the limit *β* → ∞ of high intensity of choice. Then $\sigma_{\beta }\left(P_{1}^{\left(1\right)}-P_{2}^{\left(1\right)}\right)$ approaches one if the payoff at the first market $P_{1}^{\left(1\right)}$ is larger than at the second, otherwise zero. Where the payoffs are equal, a step in the curve results, which will always produce an intersection and hence a self-consistent solution. Because of the payoff equality, such solutions correspond exactly to potentially heterogeneous Nash equilibria (see Eq (10)). Here this type of Nash equilibrium is realized in a *homogeneous mixed* form: all players from class 1 play the same strategy, choosing market 1 with probability ${\bar{p}}^{\left(1\right)}$.

If the payoffs $P_{1}^{\left(1\right)}$ and $P_{2}^{\left(1\right)})$ are different across the entire range of ${\bar{p}}^{\left(1\right)}$, we have a different scenario: assuming $P_{1}^{\left(1\right)}>P_{2}^{\left(1\right)}$ for definiteness, $\sigma_{\beta }\left(P_{1}^{\left(1\right)}-P_{2}^{\left(1\right)}\right)$ tends to one for *β* → ∞, hence the only self-consistent solution is ${\bar{p}}^{\left(1\right)}=1$.

This corresponds to a *homogeneous pure* Nash equilibrium, with—because of the assumed symmetry—the two classes of players trading at different markets.

**Fig 7:**
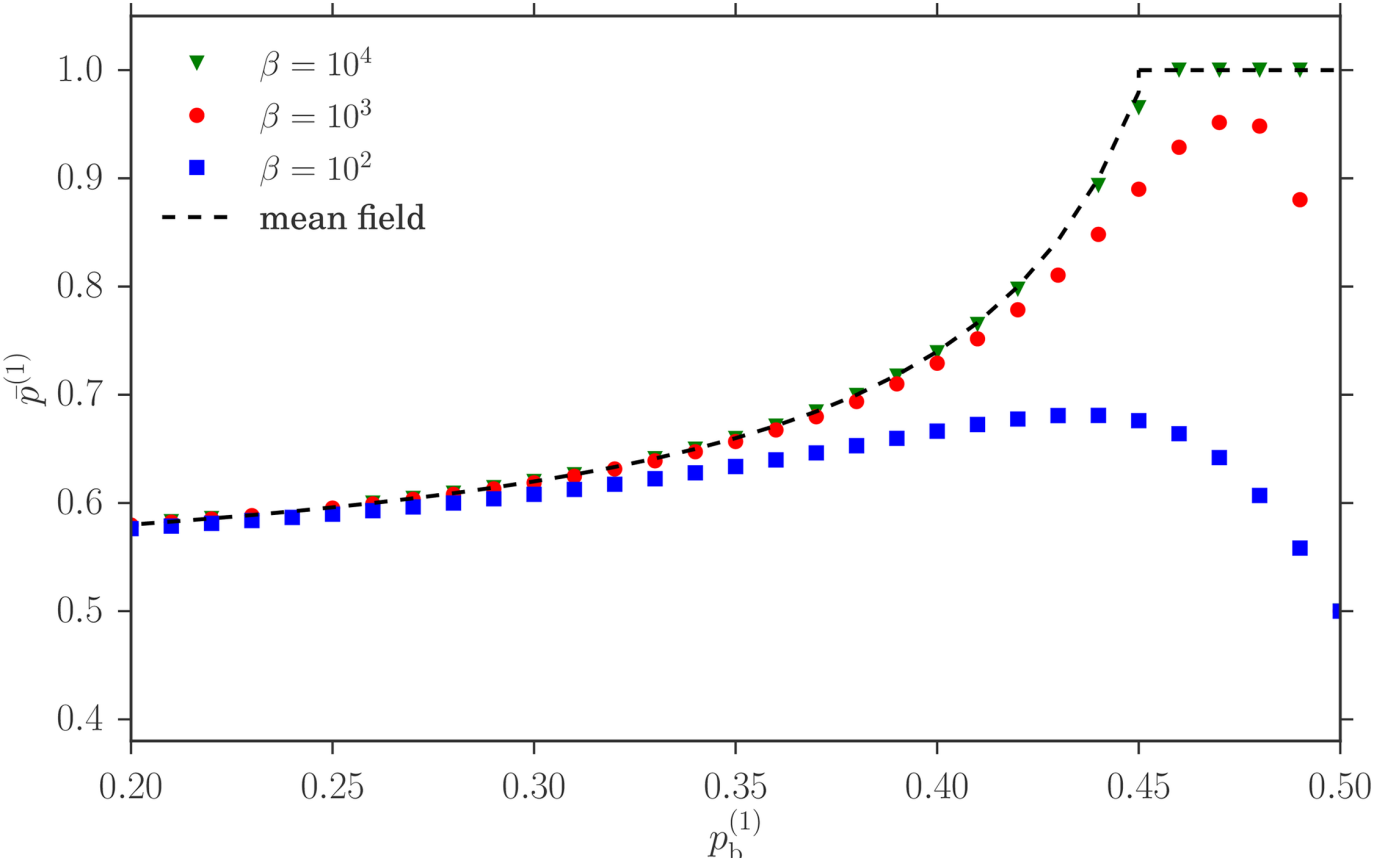
Comparison between mean field Nash equilibria (continuous lines) and homogeneous steady states of our reinforcement learning (symbols) for three different values of the intensity of choice *β*. The market biases are *θ*_1_ = 1 − *θ*_2_ = 0.3 and the buying probabilities $p_{\text{b}}^{\left(1\right)}=1-p_{\text{b}}^{\left(2\right)}=p_{\text{b}}$. Shown is ${\bar{p}}^{\left(1\right)}$, the fraction of traders from the first class going to the first market, versus $p_{\text{b}}^{\left(1\right)}$.

To show the approach to the large *β*-limit, we show in Fig 7 numerically determined values of ${\bar{p}}^{\left(1\right)}$, the fraction of traders from the first class going to the first market in the steady state of the dynamics of our model. The results for three different *β* are compared to the values of ${\bar{p}}^{\left(1\right)}$ determined from the mean field Nash equilibrium condition, which as we saw leads to the two payoff equalities (10). As expected, as *β* gets larger, the aggregate ${\bar{p}}^{\left(1\right)}$ gets closer to its Nash equilibrium value, confirming our reasoning above. Note around $p_{\text{b}}^{\left(1\right)}=0.45$ we transition from the situation in Fig 2(a), where the Nash equilibrium and the corresponding steady state are of homogeneous mixed type (green triangle in the figure), to the homogeneous pure state (orange square) in Fig 2(b).

So far our main conclusion is that steady states of our reinforcement learning model can give *homogeneous mixed* realizations of the potentially heterogeneous Nash equilibria we had identified in the results section: even though the equilibrium could be heterogeneous, the dynamics generates a homogeneous steady state with the same aggregates where all players use the same mixed strategy. This happens if we consider the limit of the dynamics for *β* → ∞ at *α* = 0. One would expect from the phase diagram in Fig 5 that the same steady state is obtained if we move the path of approach towards (*α*, 1/*β*) = (0, 0) slightly away from the vertical axis, *i.e*. if *α* is nonzero but goes to zero sufficiently fast as *β* grows. We show in the methods section that this is true if the decay of *α* is exponential, *α*_*c*_ ∼ exp(−*const* ⋅ *β*): if the constant in the exponent is large enough, the attraction distributions remain homogeneous and attractions again become equal to payoffs for *β* → ∞.

### Heterogeneous attraction distributions

We investigate in this section steady states of our reinforcement learning where the attraction distributions of traders are multimodal (heterogeneous) rather than unimodal. As explained in the results section, for *r* → 0 the modes become sharp peaks so that unimodal distributions become homogeneous. We have investigated the latter case so far, but heterogeneous steady states should also exist. Indeed, it was shown in [6] using multi-agent simulations as well as theoretical studies of the Kramers-Moyal expansion detailed in the methods section that for high enough intensity of choice *β* the distribution of attractions undergoes a transition from homogeneous to heterogeneous. We therefore expect to find heterogeneous steady states of our reinforcement learning more generally for large *β* and *α* not too small. We confirm this expectation in this section, where we also find surprising transitions between different types of heterogeneous steady states.

#### Difference between the case of homogeneous and heterogeneous attraction distributions

In [6], Alorić *et al*. describe a method to obtain the critical *α* at which the attraction distributions of the traders in the two classes become heterogeneous. One assumes initially that the distributions are homogeneous and determines a self-consistent assignment of the aggregates ${\bar{p}}^{\left(1\right)}$, ${\bar{p}}^{\left(2\right)}$ on this basis. One then checks whether the single agent dynamics for these aggregate values has one fixed point, producing a homogeneous distribution of attractions, or two or more (stable) fixed points, giving a heterogeneous distribution with peaks at these locations in attraction space. What this method leaves open, however, is what the weights of these peaks are and in particular whether they remain nonzero in the large memory limit *r* → 0. This is the task we tackle using large deviation methods, as summarized in the results section above and described in more detail in the methods section.

#### Transition from one to two to three stable fixed points

We next explore the different fixed point structures of the single agent dynamics as a function of the fictitious play parameter *α*, for fixed large intensity of choice *β*. In principle at each *α* the aggregates ${\bar{p}}_{1}^{\left(c\right)}$, ${\bar{p}}_{2}^{\left(c\right)}$ need to be determined from self-consistency but from the experience with the homogeneous solutions we expect that as long as *α* is small enough and *β* large enough, the self-consistent aggregate values will be close to their Nash equilibrium values. To leading order one can therefore think of varying *α* at fixed aggregates. As before we also rely on the assumption that the memory of the traders is large (*r* → 0); the finite memory case will be investigated below using numerical simulations.

**Fig 8:**
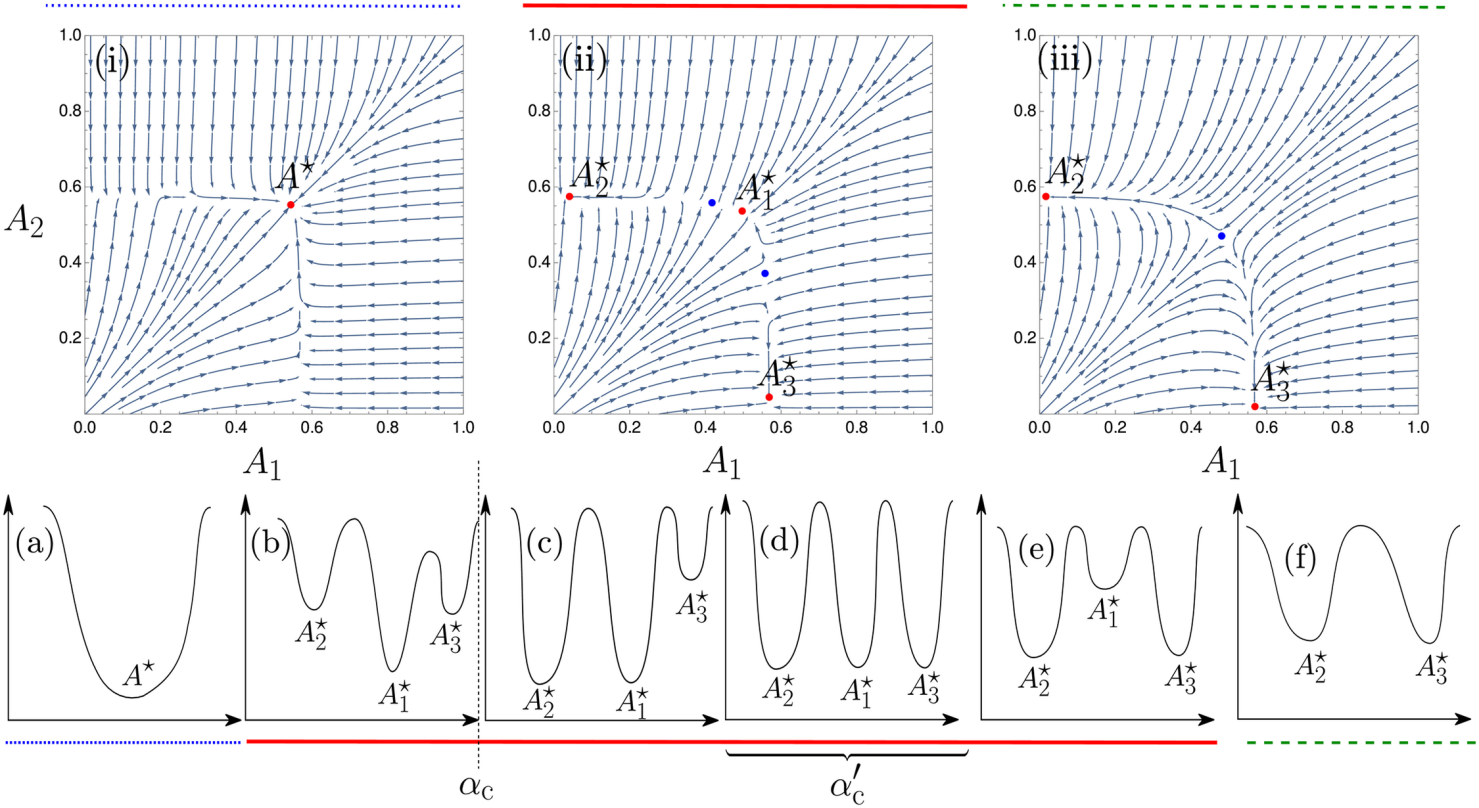
(i-iii) Flow diagrams of the single agent dynamics for increasing *α*. The points represent the stable (red) and unstable (blue) fixed points of the dynamics. The potentials in the bottom row represent schematically in 1-D the arrangement of fixed points (stable = potential minimum, unstable = potential maximum). Attraction distributions are peaked around stable fixed points; in the 1-D representation, the lowest minima indicate peaks with weights of order unity as *r* → 0, while higher-lying (metastable) minima correspond to peaks that become exponentially suppressed. For *α* < *α*_*c*_, the aggregates of the single agent dynamics are deduced by self consistency from the only stable fixed point of the dynamics (panels (a) and (b)), while for larger *α* the aggregates are chosen such that the transition rates between the stable fixed points ($A_{1}^{⋆}$ and $A_{2}^{⋆}$ for $\alpha_{c}<\alpha <\alpha_{\text{c}}^{\prime }$, panels (c) and (d); $A_{1}^{⋆}$ and $A_{3}^{⋆}$ for $\alpha >\alpha_{\text{c}}^{\prime }$, panels (e) and (f)) are of the same order. Plots were produced with symmetric market biases *θ*_1_ = 1 − *θ*_2_ = 0.3 and probability of buying $p_{\text{b}}^{\left(1\right)}=1-p_{\text{b}}^{\left(2\right)}=0.2$ and intensity of choice *β* = 1/0.11.

When the fictitious play coefficient *α* is small enough, the single agent dynamics has a single stable fixed point $A_{1}^{⋆}$ (see the Methods section and Fig 8(i)) and so for *r* → 0 the distribution of attractions is a *δ*-peak at this point as shown in Fig 3(a). As *α* increases then as shown in Fig 8(b) two new stable fixed points $A_{2}^{⋆}$ and $A_{3}^{⋆}$ appear, first one and then the other. But the distribution of attractions is still delta peaked around the original fixed point because in the limit *r* → 0 the other fixed points are exponentially suppressed in 1/*r*: they are in this sense metastable.

The first phase transition arises at a critical value of *α*, *α*_*c*_, where one of the metastable point becomes stable; in Fig 8 this is $A_{2}^{⋆}$. In this case, the attraction distribution is composed of two *δ*-peaks located at these two stable fixed points of the single agent dynamics (see Fig 3(a) and 3(b) for an example projected onto one direction in attraction space). The transition occurs because the actions (see the Methods section) for single agents to move from one stable fixed point to the other and for the reverse move become equal.

This ensures that the fluxes of agents between the two stable fixed points are of the same order of magnitude in both directions, and hence that the two peaks in the attraction distribution can have comparable rather than exponentially different weights.

As *α* increases further, small changes to the aggregates maintain the condition of comparable flux between the two existing stable peaks. Eventually, at some $\alpha_{\text{c}}^{\prime }$ higher than *α*_*c*_, the third fixed point also becomes stable so that the attraction distribution acquires three peaks.

Note that the weights of the three peaks cannot be fully determined at $\alpha =\alpha_{\text{c}}^{\prime }$: the self-consistency for ${\bar{p}}^{\left(1\right)}$ only gives one condition for three nonnegative peak weights that need to sum to one, so that the problem is underconstrained. This indicates that for nonzero *r* these weights would vary continuously across a small range of *α* of order *r*.

For $\alpha >\alpha_{\text{c}}^{\prime }$, it is the turn of the central fixed point $A_{1}^{⋆}$ to become metastable; aggregate values are determined by the equal action condition between the two outer stable fixed points and the attraction distribution goes back to having only two *δ*-peaks. Finally at even larger *α* the central metastable fixed point disappears altogether in a saddle-node bifurcation.

#### Game theoretical interpretation of the steady states

We now investigate the characteristics of all the steady states described above and compare each of them to the Nash equilibria enumerated in the results section. When *α* is below the critical value *α*_*c*_, all the traders within one class randomize between the two markets, going to the first market with the same probability. This probability is $\sigma_{\beta }\left(A_{1}^{\left(c\right)}-A_{2}^{\left(c\right)}\right)$ evaluated at the stable fixed points of the single agent deterministic dynamics, which also equals ${\bar{p}}^{\left(c\right)}$ (see the Results section). This *homogeneous mixed* strategy profile is plotted as the single-peaked preference distribution in Fig 3.

For the opposite case of large *α*, $\alpha >\alpha_{\text{c}}^{\prime }$, there are within each class two sub-populations of traders, each of which corresponds to a peak of the attraction distribution as shown schematically in Fig 3(b). Looking at Fig 8(iii) and 8(f), one sees that at both of these peaks, the attractions to the two markets remain distinct for large *β*—the relevant fixed points are far from the 45° diagonal. In the limit both sub-populations will therefore play a pure strategy as $\sigma_{\beta }\left(A_{1}^{\left(c\right)}-A_{2}^{\left(c\right)}\right)$ tends to one or zero, respectively. This situation is shown as the preference distribution in Fig 3(b) with two peaks around preference one and zero, representing two sub-populations of traders all choosing market 1 and 2 respectively. This steady state of our reinforcement learning model is therefore a *heterogeneous pure* realization of a Nash equilibrium, as the preferences of traders are heterogeneous, with two sub-population playing different pure strategies.

While the two cases of homogeneous mixed and heterogeneous pure Nash equilibria are well studied in the literature [20, 21], we find a novel state for *α*_*c*_ < *α* < *α*_c_′. Again there are within each class two sub-populations of traders. But now one sub-population has attractions that become equal for large *β*: the corresponding fixed point lies close to the diagonal in Fig 8(ii). These traders therefore play a mixed strategy and randomize between the two markets. Overall we have a *heterogeneous mixed* steady state because not all traders play pure strategies. This is illustrated in the right panel of Fig 3. Such heterogeneous mixed strategy distributions have, to our knowledge, never been reported in any study of aggregative games so it is fascinating that they are accessible by our variation of EWA learning dynamics.

Overall, we have found that potentially heterogeneous Nash equilibria can be realized as steady states of our variation of EWA learning in three different ways by appropriately taking the limits of perfect fictitious play *α* → 0 and best response *β* → ∞. For small enough *α* < *α*_c_(*β*) one obtains a homogeneous mixed equilibrium, while keeping larger $\alpha >\alpha_{\text{c}}^{\prime }\left(\beta \right)$ gives a heterogeneous pure equilibrium. Most interesting is the case where *α* is taken to zero in the “corridor” $\alpha_{\text{c}}<\alpha <\alpha_{\text{c}}^{\prime }$, which results in a heterogeneous mixed equilibrium.

Note that the partially heterogeneous Nash equilibria (where one class of traders splits into sub-populations while the other stays homogeneous) do not appear in the analysis above because we restricted ourselves to studying Nash equilibria for which the aggregates are symmetric (${\bar{p}}^{\left(1\right)}=1-{\bar{p}}^{\left(2\right)}$), thus ruling out partially heterogeneous Nash equilibria.

**Fig 9:**
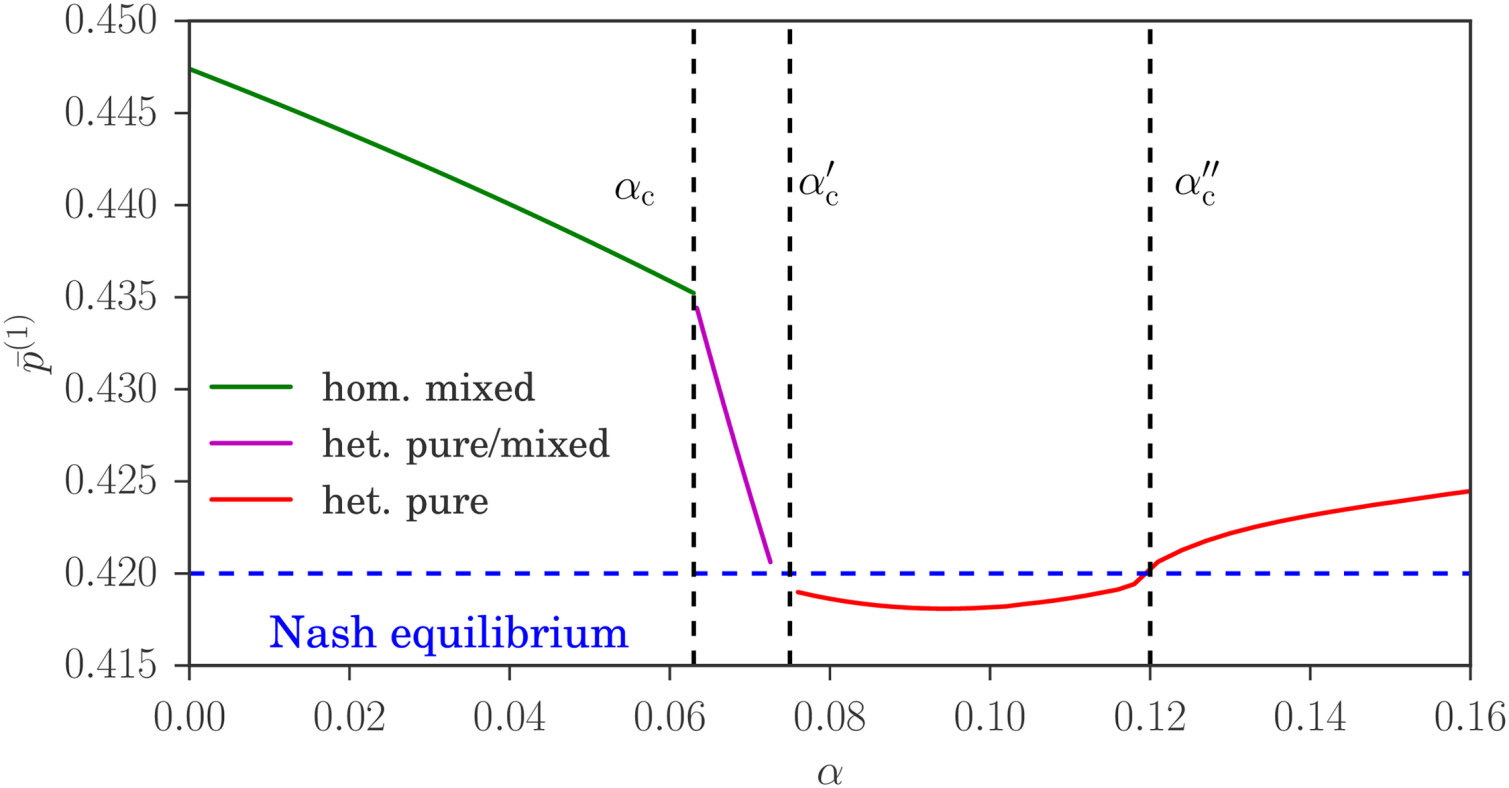
Fraction of traders from the first class in the first market, ${\bar{p}}_{1}^{\left(1\right)}$, for intensity of choice *β* = 1/0.11, compared with the value of ${\bar{p}}^{\left(1\right)}$ calculated for the corresponding potentially heterogeneous Nash equilibrium (see the Results section). Note that the deviation between the two values is small throughout. Critical values of *α* separating the different types of steady states are indicated; $\alpha_{\text{c}}^{\prime \prime }$ is the value of *α* where the “central” fixed point representing traders playing mixed strategies disappears. Same system parameters as in Fig 8.

We close this section by showing in Fig 9 some numerical results for the aggregate ${\bar{p}}^{\left(1\right)}$ as a function of *α*, for a fixed intensity of choice *β*. The values of *α*_c_ and $\alpha_{\text{c}}^{\prime }$ are shown to indicate the transitions between the homogeneous mixed, heterogeneous mixed and heterogeneous mixed states as *α* grows. Also shown is the even larger critical value $\alpha_{\text{c}}^{\prime \prime }$ at which the “central” fixed point (see Fig 8) disappears. Note the vertical scale of the plot, which demonstrates a key point: even though *β* = 1/0.11 is not yet very large, ${\bar{p}}^{\left(1\right)}$ is already quite close to the value ${\bar{p}}^{\left(1\right)}\approx 0.42$ for the potentially heterogeneous Nash equilibrium as calculated using the equal payoff criterion (10) in the results section.

**Fig 10:**
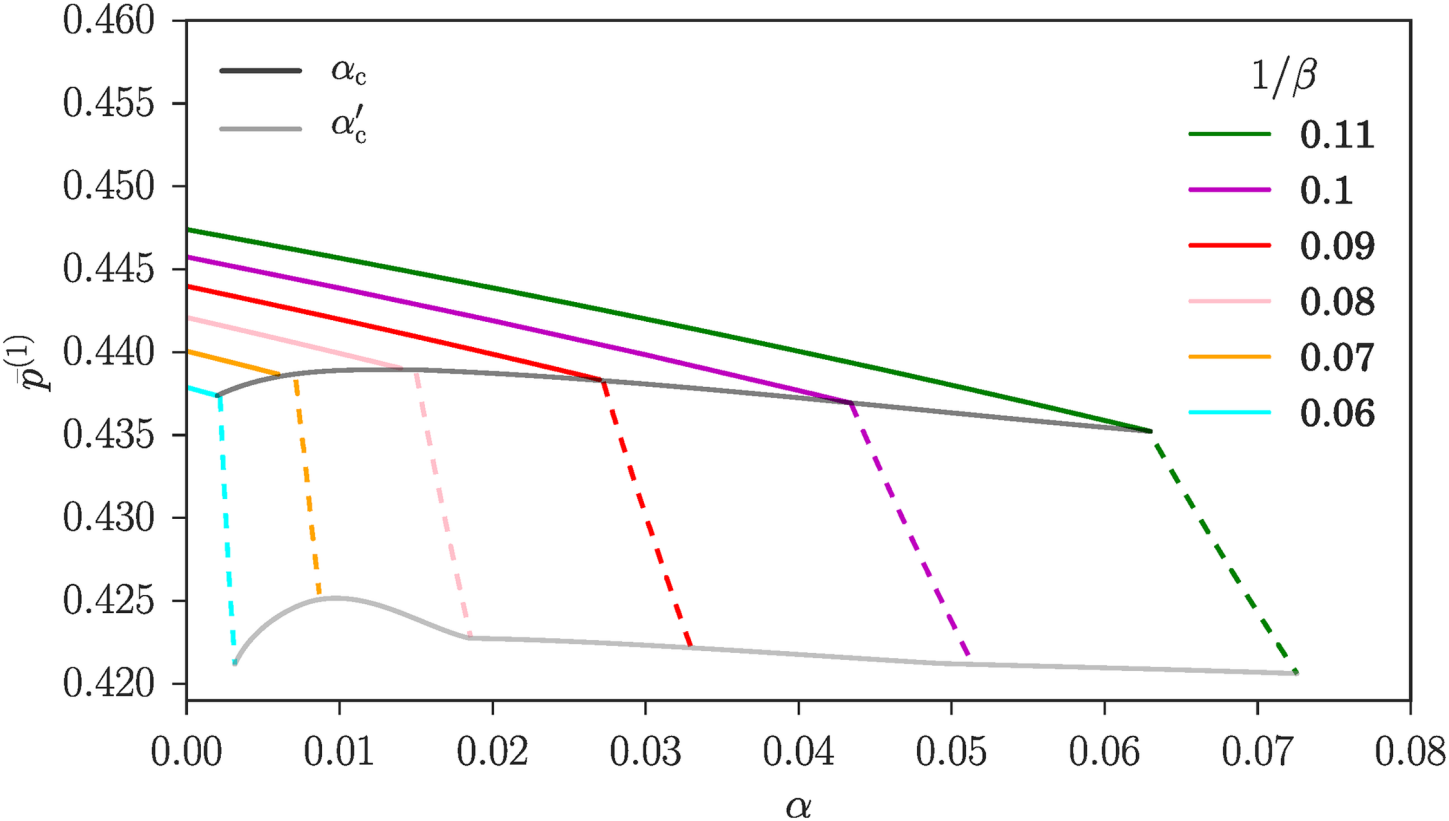
Fraction of players from class 1 in the first market, ${\bar{p}}^{\left(1\right)}$, for different values of *β*. The grey lines connect the values at the two critical *α* (see Fig 8) as a guide to the eye. System parameters as in Fig 8. Note that ${\bar{p}}^{\left(1\right)}$ gets progressively closer to the Nash equilibrium value ≈ 0.42 as the intensity of choice *β* grows.

As we have argued this agreement should get even better as *β* grows. Numerical data supporting this are shown in Fig 10: ${\bar{p}}^{\left(1\right)}$ decreases towards the Nash equilibrium value with increasing *β*. Also displayed are the critical values *α*_c_ and $\alpha_{\text{c}}^{\prime }$, which as expected tend to zero as *β* grows. It is these values that were used to produce the phase diagram in Fig 5.

We note as an aside that in Fig 10 the variation of ${\bar{p}}^{\left(1\right)}$ with *α* is rather steeper in the heterogeneous mixed phase (between *α*_c_ and $\alpha_{\text{c}}^{\prime }$) than in the homogeneous mixed regime. This probably reflects the change in the way the aggregates are determined in the two regimes: in the homogeneous-mixed phase the aggregates are obtained only by the self-consistency condition for the fixed point location, while they are fixed by the equal flux condition in the heterogeneous mixed phase.

#### Test against simulations

In this section we test the theoretical predictions obtained above in the *r* → 0 and for infinite population size *N* against agent based simulations with a finite memory (*r* > 0) and finite *N*. We are primarily interested in the steady state of the attraction distribution of the agents, but also consider its time evolution to this steady state. We continue to consider symmetric scenarios so focus on the properties of agents of class 1 throughout. Depending on where the key parameters *α* and *β* are in the phase diagram of Fig 5, one expects qualitatively different shapes for the attraction distribution resulting from the learning dynamics. We present simulation results in each of the distinct regions of the phase diagram in Fig 5.

**Fig 11:**
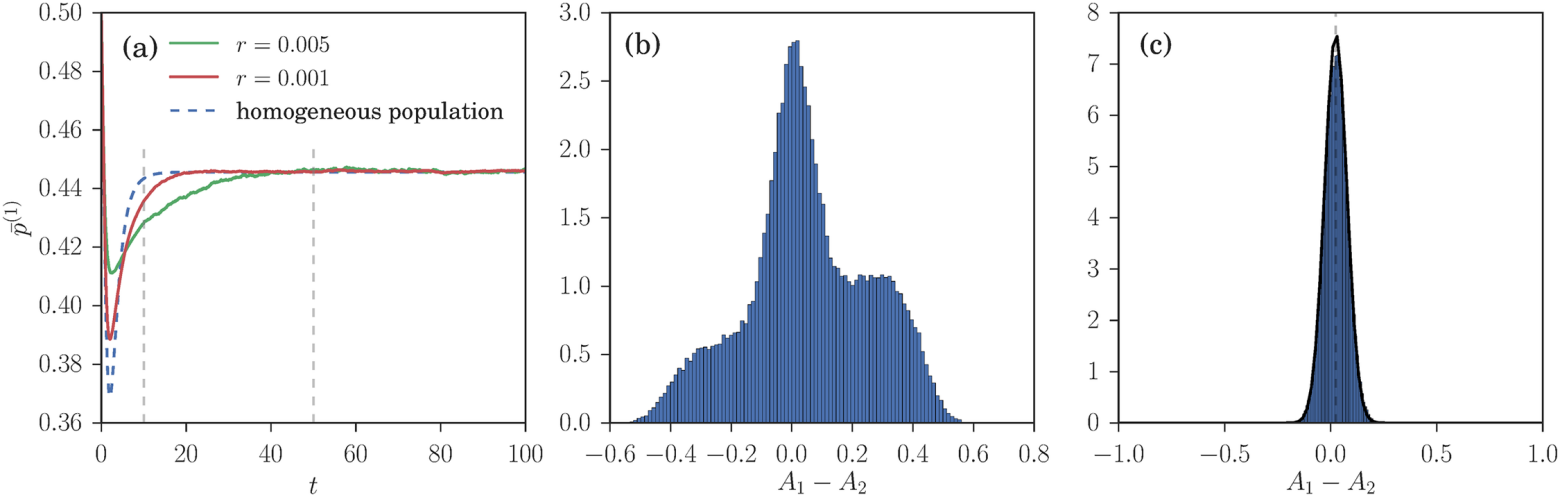
Reinforcement learning dynamics at small *α* = 0.01. (a) Time evolution of ${\bar{p}}^{\left(1\right)}$ for *r* = 0.005 and *r* = 0.001 compared to the homogeneous population dynamics predicted for (*r* → 0). (b, c) Distribution of attraction differences across traders of class 1 at two times, for *r* = 0.005. Black lines are theoretical predictions based on the homogeneous population dynamics and agree well at small *r* and late times *t* as expected (see text). Note that for the larger *r*, the dynamics (a) and the attraction distributions (b) deviate from the small-*r* theory, showing a transient spontaneous emergence of preferences heterogeneity that is the precursor of steady state preferences heterogeneity (see Fig 13(d)) at larger *α*. The parameters used for those simulation are *β* = 1/0.11, *θ*_1_ = 1 − *θ*_2_ = 0.3, $p_{\text{b}}^{\left(1\right)}=1-p_{\text{b}}^{\left(2\right)}=0.2$, the system is composed of 20000 traders.

The first zone of interest is on the far left of the phase diagram, where *α* is below the first threshold *α*_*c*_. Here, in the steady state of the learning dynamics, we observe in Fig 11(c) the homogeneous distribution of preference predicted by the theory. Looking beyond this agreement for the steady state at the time evolution, panel 11(a) shows that for *r* = 0.005 the transient dynamics of the aggregates is nonetheless different from the homogeneous population deterministic dynamics. This appears to be related to a transient preferences heterogeneity observed in a small time window around *t* = 10 (Fig 11(b)). This transient spontaneous emergence of preferences heterogeneity does not occur for lower values of *r* (*e.g*. *r* = 0.001), where the dynamics of the aggregates is closer to the homogeneous population dynamics (see Fig 11(a)).

**Fig 12:**
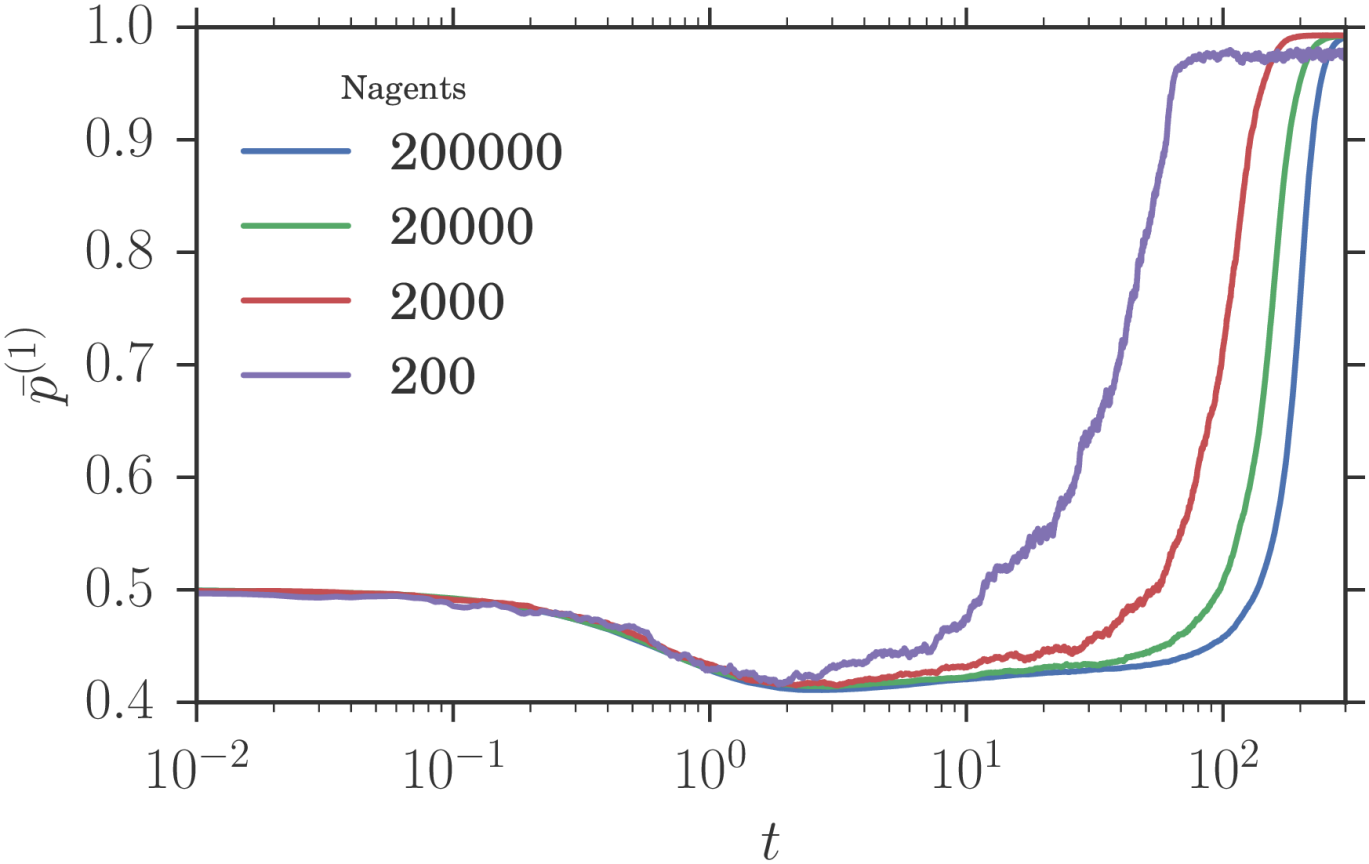
Time evolution of ${\bar{p}}^{\left(1\right)}$ for *α* = 0.068, *r* = 0.005 and different numbers of agents *N*. Other parameters are the same as in Fig 8.

**Fig 13:**
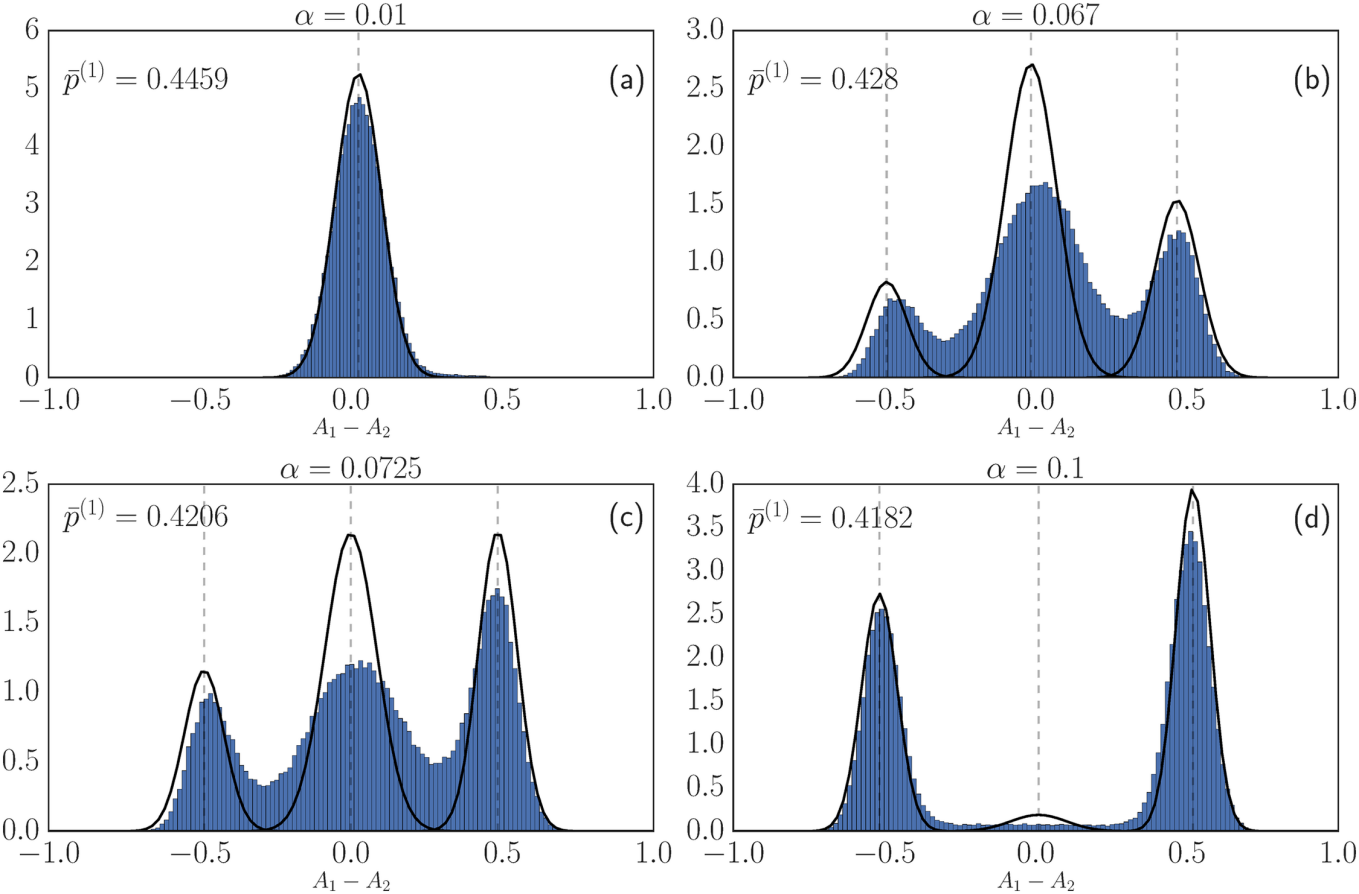
Steady state distribution of the attraction differences for *r* = 0.01, and increasing values of *α*; the remaining parameters are as in Fig 8. When *α* = 0.067 (panel (b)), the theory predicts one outer peak on the right and one inner peak corresponding to a fraction of the population playing a mixed strategy. The simulations additionally show an outer peak on the left, which arises from the fact that the finite-*N* system is not in a true steady state. Panel (c) shows the situation for *α* = 0.0725, which is the critical value $\alpha_{c}^{\prime }$ at which we expect to see from theory three different peaks in the distribution of attraction differences. The theoretical predictions (black curves) is a Gaussian mixture composed of three peaks whose mean and variance are obtained from the Kramers-Moyal expansion while their weights, which the theory cannot predict, are fitted to the data. The peak positions are in good agreement with theory while the simulations overestimate the variance of the peaks, again because of transient effects. In panel (d), for $\alpha >\alpha_{c}^{\prime }$, there is very good agreement with theory except for a small central peak that for *r* → 0 is predicted to have weight zero. This is likely to be an effect of the nonzero *r* = 0.01 used in the simulations.

When $\alpha \in [\alpha_{c},\alpha_{\text{c}}^{\prime }]$, the aggregates relax close to their value in a Nash equilibrium around which they fluctuate. Then, they escape from this state to reach an heterogeneous pure Nash equilibrium. The time they remain close to the Nash equilibria depends on the number of agents in the simulation as shown in Fig 12. The theory predicts a distribution composed of two peaks, one peak corresponding to a sub-population playing mixed strategies and the second one to a sub-population playing pure strategies. The results of our simulation presented in Fig 13(a) show a preference distributions composed of three peaks, not two as the theory predicts. One also notices that while the theoretical predictions for the location of the peaks are consistent with the simulation results, the width of the peaks in the simulations is larger than predicted. We believe this is because the theoretical predictions for the width of the peaks make the assumption that the system is in its steady state. This is not strictly verified here as the finite-*N* system is in a transient state before relaxing to a heterogeneous pure distribution of strategies. As *α* goes above the SEHP threshold, $\alpha_{\text{c}}^{\prime }$, the dynamics initially continues to show three peaks, but in qualitative agreement with the theory the size of the central peak diminishes rapidly, becoming negligible for large enough *α*. The preference distributions obtained from simulations are then consistent with the theoretical predictions as shown in Fig 13(d). Moreover, the aggregates stay close to *f*_1_ = 0.42 and never diverge to *f*_1_ = 1 or *f*_1_ = 0 (as happens for lower values of *α*).

In summary, the simulations are in good qualitative accord with the predicted sequence of steady states for increasing *α*: homogeneous mixed, heterogeneous mixed (outer and central peak), heterogeneous mixed (three-peaked) and finally heterogeneous pure (two outer peaks). Corrections to the theoretical predictions arise from the fact that some steady states have a lifetime that only becomes infinite for *N* → ∞, and from the use of nonzero *r* in the simulations.

## Methods

### Large deviation

We describe in this section the large deviation methods we use to study heterogeneous attraction distributions in the steady state of our reinforcement learning model. As explained in the results section, steady state attraction distributions for small *r* will be peaked around the stable fixed points of the single agent dynamics. The shape of these peaks becomes Gaussian for *r* → 0, with a covariance matrix proportional to *r* that is straightforward to determine. Much more difficult to find are the *weights* of the peaks as these involve rare fluctuations of an agent making the transition from one peak to another. In one dimension the problem is tractable as an explicit formula for the steady state distribution of attractions can be given [6]. In higher dimensions detailed balance [29] has a similar simplifying effect, but our single agent dynamics in the two-dimensional attraction space (for each class of agents) does not have this property.

In our approach we consider the peak weights in an attraction distribution as a result of the balance between transitions between the various peaks. We therefore need to find the rates for these transitions. To do this, note from the Kramers-Moyal expansion that the single agent reinforcement learning is described by a Langevin equation with noise variance *O*(*r*). For *r* → 0 we are therefore looking for transition rates in a low noise limit. This allows us to use Freidlin-Wentzell theory, which deals the with large deviations of Langevin dynamics in exactly this limit [30].

#### Freidlin-Wentzell theory

We use Freidlin-Wentzell theory in the form developed in [31, 32], which generalizes the Eyring-Kramers [33] formula for the rates of noise-activated transitions to non-conservative dynamics such as our reinforcement learning dynamics. We give a brief summary of those aspects of Freidlin-Wentzell theory that we use in our numerical application and refer to [30] for a mathematically rigorous description and to [31] for a more statistical physics-oriented summary.

Freidlin-Wentzell theory is concerned with the transition rates between two stable states (here $A_{1}^{⋆}$ and $A_{2}^{⋆}$) of a non-conservative stochastic dynamics in the low noise limit. A general Langevin equation can be written in the form

$$\begin{array}{c} {\dot{A}}^{\left(c\right)}\left(t\right)=\mu^{\left(c\right)}\left(A^{\left(c\right)}\left(t\right),{\bar{p}}^{\left(1\right)},{\bar{p}}^{\left(2\right)}\right))+\sqrt{r}{\Sigma^{\left(c\right)}}^{1/2}\left(A^{\left(c\right)}\left(t\right),{\bar{p}}^{\left(1\right)},{\bar{p}}^{\left(2\right)}\right))\xi \left(t\right) \end{array}$$ (22)

where **ξ**(*t*) is white noise with unit covariance matrix. The drift ***μ*** and the covariance matrix **Σ** of the noise in the Langevin equation are given in Eq (47) for our specific variation of EWA learning, where the Langevin description results from a second order Kramers-Moyal expansion (46). In the generic version above we have omitted the superscript (*c*) indicating the class of agents we are considering, as well as the dependence of drift and noise covariance on the aggregates ${\bar{p}}^{\left(1\right)}$ and ${\bar{p}}^{\left(2\right)}$.

Associated with the Langevin dynamics is an Onsager-Machlup action S[A] for any path ***A***(*t*):

$$\begin{array}{c} S\left[A\right]=\int_{t_{1}}^{t_{2}}\frac{1}{2}{\left(\dot{A}\left(t\right)-\mu \left(A\left(t\right)\right)\right)}^{T}\Sigma^{-1}\left(A\left(t\right)\right)\left(\dot{A}\left(t\right)-\mu \left(A\left(t\right)\right)\right)\text{d}t \end{array}$$ (23)

The action determines the probability of observing any path [***A***(*t*)] according to

$$\begin{array}{c} \Gamma_{1\to 2}∼\exp\left(-S\left[A\right]/r\right) \end{array}$$ (24)

where ∼ means that the equality is true up to a pre-factor (which depends on the time discretization used).

The main Freidlin-Wentzell result we need is that the rate Γ_1 → 2_ for a transition from $A_{1}^{⋆}$ to $A_{2}^{⋆}$ (*forward path*) is [30, 34]

$$\begin{array}{c} \Gamma_{1\to 2}∼\exp\left(-S_{1\to 2}^{⋆}/r\right) \end{array}$$ (25)

where $S_{1\to 2}^{⋆}$ is the minimal action achievable by any paths from $A_{1}^{⋆}$ to $A_{2}^{⋆}$ in the infinite time interval (*t*_1_, *t*_2_) = (−∞, ∞). The rate Γ_2 → 1_ for the *reverse* transition from $A_{2}^{⋆}$ to $A_{1}^{⋆}$ is similarly $\Gamma_{2\to 1}∼\exp\left(-S_{2\to 1}^{⋆}/r\right)$.

The attraction distributions we are after will consist of narrow (for small *r*) peaks at $A_{1}^{⋆}$ and $A_{1}^{⋆}$. The weights *ω*_1_ and *ω*_2_ of these two peaks, which represent the probability for an agent to be within each peak, must then be such that forward and backward transitions balance:

$$\begin{array}{ccc} \omega_{1}\Gamma_{1\to 2} & = & \omega_{2}\Gamma_{2\to 1} \end{array}$$ (26)

$$\begin{array}{ccc} \frac{\omega_{1}}{\omega_{2}} & \propto & \exp(\frac{S_{1\to 2}^{⋆}-S_{2\to 1}^{⋆}}{r}) \end{array}$$ (27)

This expression shows that when the forward and backward minimal actions are not equal, then one of the two peaks will have an exponentially small weight as *r* → 0. In practice this is true when the action difference inside the exponential in (26) is large compared to *r*. If it is only of order *r* or smaller, then we cannot say anything about the weights as we do not determine prefactor in (26), though we would expect them to be of order unity.

#### Finding the minimal action path numerically

Following the method of Bunin *et al*. [34], we find the minimal action by discretizing the path [**A**(*t*)], evaluating the action as a function of this discretized path and then minimizing with respect to the (discretized) path. The path is discretized into 10 equally spaced timesteps between *t* = 0 and *t* = 10; we found this choice of parameters to be a reasonable trade-off between the precision of our result and the complexity of minimizing the discretized action.

There are other methods for finding the minimal value of the action defined in Eq (23), such as solving a Hamilton-Jacobi equation [31], but we chose to use the path discretization method because we found this to be more robust with respect to changes of model parameters. The discretization approach could also be improved further, using for example the geometric minimum action method [35], but we found that this was not necessary to achieve the desired precision. We tested this e.g. by benchmarking against closed-form results that can be obtained for *α* = 1 [6].

**Fig 14:**
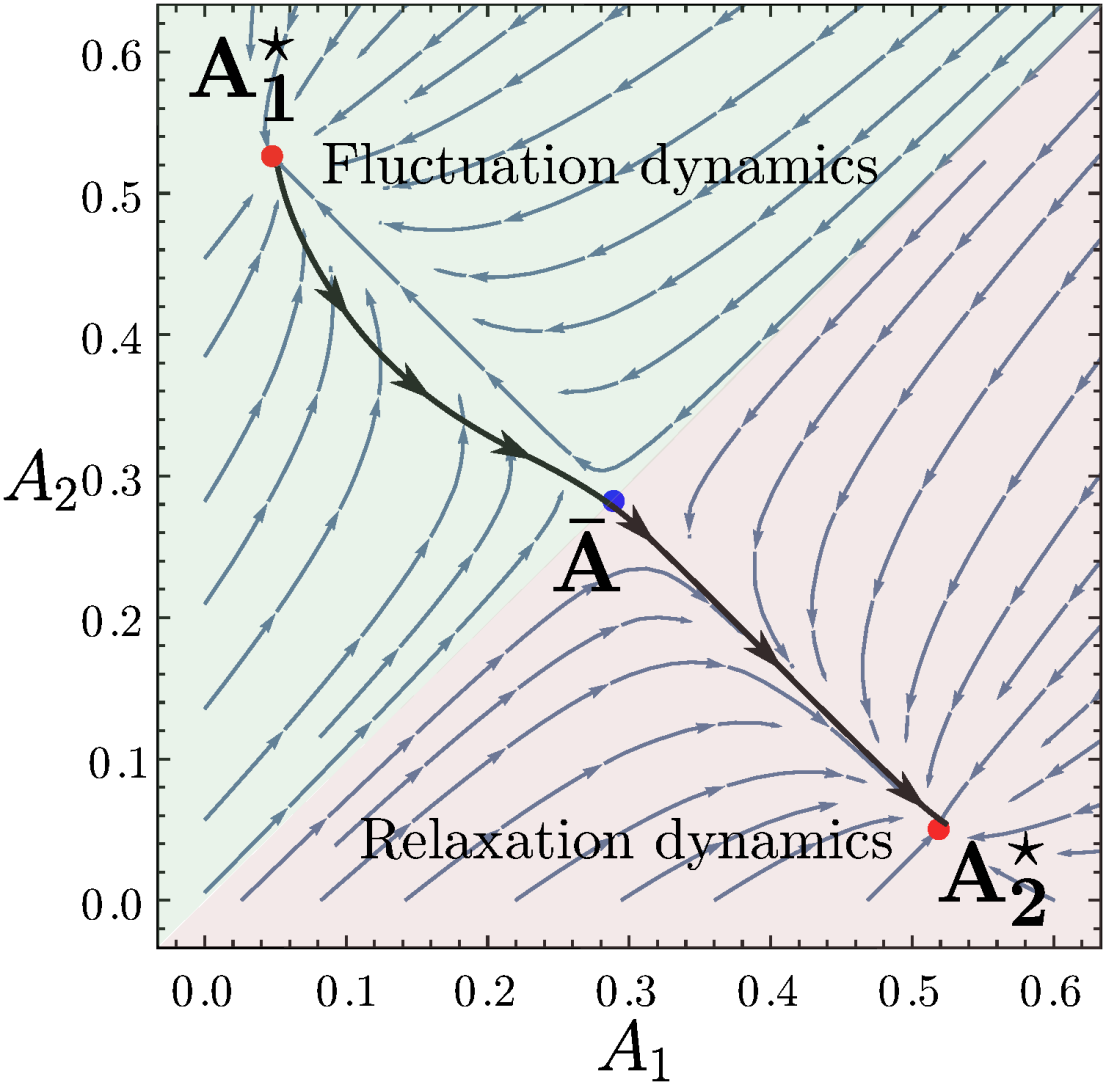
An example of a minimal action path, from fixed point $A_{1}^{⋆}$ to $A_{2}^{⋆}$. The path starts with a “fluctuation” (or: activation) segment that ends at the saddle point $\bar{A}$ between the two fixed points. The remainder of the path is a “relaxation” segment that follows the deterministic dynamics and incurs zero contribution to the action.

The numerical path optimization can be simplified by restricting attention to the *activation* part of the path. Generally, for a system with two stable fixed points $A_{1}^{⋆}$ and $A_{2}^{⋆}$ and one saddle point $\bar{A}$ between them, the optimal path starting from $A_{1}^{⋆}$ will pass through the saddle point $\bar{A}$ and then relax to $A_{2}^{⋆}$ following the relaxation dynamics $\dot{A}\left(t\right)=\mu \left(A\left(t\right)\right)$, as sketched in Fig 14 [30]. Eq (23) shows that the relaxation dynamics does not contribute to the total action as the integrand (the Lagrangian) vanishes identically along this section of the path. As a consequence, the problem of finding a minimal action path between $A_{1}^{⋆}$ and $A_{2}^{⋆}$ can be reduced to finding the minimal action path between $A_{1}^{⋆}$ and $\bar{A}$, *i.e*. from the initial fixed point to the saddle. This restriction significantly improves the precision of the numerical path optimization.

With the above method, we can work out the action difference between any two fixed points of the single agent dynamics, as a function of the aggregates ${\bar{p}}^{\left(1\right)}$, ${\bar{p}}^{\left(2\right)}$; only the first of these is needed for symmetric steady states. The values of ${\bar{p}}^{\left(1\right)}$ where the action difference between two single agent fixed points vanishes identify the points where the steady state attraction distribution of our reinforcement learning dynamics can have more than one peak. Either side of these values, a single peak is dominant in the attraction distribution; which peak this is changes discontinuously at a zero action difference value of ${\bar{p}}^{\left(1\right)}$, see Fig 6.

### Formula for the payoff

To work out the average payoff of an ask (a) or bid (b) at market *m*, we find first the probability for such an order to be valid:

$$\begin{array}{c} V\left(\text{a},m\right)=P\left(\text{ask}\;\text{price}\;<\pi_{m}\right)=\frac{1}{\sqrt{2\pi }\sigma }\int_{-\infty }^{\pi_{m}}\exp\left(-\frac{{\left(x-\mu_{\text{a}}\right)}^{2}}{2\sigma^{2}}\right)\text{d}x \end{array}$$ (28)

$$\begin{array}{c} V\left(\text{b},m\right)=P\left(\text{bid}\;\text{price}\;>\pi_{m}\right)=\frac{1}{\sqrt{2\pi }\sigma }\int_{\pi_{m}}^{\infty }\exp\left(-\frac{{\left(x-\mu_{\text{b}}\right)}^{2}}{2\sigma^{2}}\right)\text{d}x \end{array}$$ (29)

where the trading price *π*_*m*_ is defined in Eq (1).

Once an order has been validated, it needs to be matched with that of a trader on the other side of the market. We denote the probability for this to happen for an order of type *τ* at market *m* by $M(\tau ,m,f_{m})$. This quantity depends on the ratio of the number of buyers and sellers in the market, $f_{m}=\frac{\text{\# buyers @ market m}}{\text{\# sellers @ market m}}$, as follows:

$$\begin{array}{c} M\left(\text{a},m,f_{m}\right)=\min\left(\frac{f_{m}V\left(\text{b},m\right)}{V(\text{a},m)},1\right) \end{array}$$ (30)

$$\begin{array}{c} M\left(\text{b},m,f_{m}\right)=\min\left(\frac{V(\text{a},m)}{f_{m}V\left(\text{b},m\right)},1\right) \end{array}$$ (31)

where the first ratio in the minimum is that of the number of *valid* buy and sell orders, always assuming large *N* where fluctuations of these numbers can be neglected.

We call $\langle S_{\tau ,m}\rangle$ the average score of an order of type *τ*, once it has been validated and successfully matched. This is given by:

$$\begin{array}{c} \left\langle S_{\text{a},m}\right\rangle =\frac{1}{V(\text{a},m)}\frac{1}{\sqrt{2\pi }\sigma }\int_{-\infty }^{\pi_{m}}\left(\pi_{m}-x\right)\exp\left(-\frac{{\left(x-\mu_{\text{a}}\right)}^{2}}{2\sigma^{2}}\right)\text{d}x \end{array}$$ (32)

$$\begin{array}{c} \left\langle S_{\text{b},m}\right\rangle =\frac{1}{V(\text{b},m)}\frac{1}{\sqrt{2\pi }\sigma }\int_{\pi_{m}}^{\infty }\left(x-\pi_{m}\right)\exp\left(-\frac{\left(x-\mu_{\text{b}}^{2}\right)}{2\sigma^{2}}\right)\text{d}x \end{array}$$ (33)

For later use we also define the average square of the score:

$$\begin{array}{c} \left\langle S_{\text{a},m}^{2}\right\rangle =\frac{1}{V(\text{a},m)}\frac{1}{\sqrt{2\pi }\sigma }\int_{-\infty }^{\pi_{m}}{\left(\pi_{m}-x\right)}^{2}\exp\left(-\frac{{\left(x-\mu_{\text{a}}\right)}^{2}}{2\sigma^{2}}\right)\text{d}x \end{array}$$ (34)

$$\begin{array}{c} \left\langle S_{\text{b},m}^{2}\right\rangle =\frac{1}{V(\text{b},m)}\frac{1}{\sqrt{2\pi }\sigma }\int_{\pi_{m}}^{\infty }{\left(x-\pi_{m}\right)}^{2}\exp\left(-\frac{{\left(x-\mu_{\text{b}}\right)}^{2}}{2\sigma^{2}}\right)\text{d}x \end{array}$$ (35)

We can now compute the average payoff of an order of type *τ* at market *m*:

$$\begin{array}{c} P_{\tau ,m}\left(f_{m}\right)=V\left(\tau ,m\right)M\left(\tau ,m,f_{m}\right)\left\langle S_{\tau ,m}\right\rangle \end{array}$$ (36)

Similarly, the average squared payoff that will appear in the second order moment of the Kramers-Moyal expansion can be expressed as

$$\begin{array}{c} Q_{\tau ,m}\left(f_{m}\right)=V\left(\tau ,m\right)M\left(\tau ,m,f_{m}\right)\left\langle S_{\tau ,m}^{2}\right\rangle \end{array}$$ (37)

$$\begin{array}{c} Q_{m}^{\left(c\right)}\left(f_{m}\right)=p_{\text{b}}^{\left(c\right)}Q_{\text{b},m}\left(f_{m}\right)+\left(1-p_{\text{b}}^{\left(c\right)}\right)Q_{\text{a},m}\left(f_{m}\right) \end{array}$$ (38)

The second version here is averaged over the preference for buying and selling of an agent in class *c*.

### Phase diagram boundaries in Fig 4

In this section we indicate how to calculate phase boundaries in Fig 4, which shows the phase diagram for the case where the market bias and the probability to buy are symmetric (*θ*_1_ = 1 − *θ*_2_, $p_{\text{b}}≐p_{\text{b}}^{\left(1\right)}=1-p_{\text{b}}^{\left(2\right)}$).

At this boundary, a (symmetric) potentially heterogeneous Nash equilibrium (green triangle in Fig 2) turns smoothly into a homogeneous pure equilibrium (blue diamond and orange square in Fig 2) where the two classes of players choose different markets. One can therefore calculate the boundary by establishing the zone in the phase diagram where this homogeneous Nash equilibrium exists. For definiteness we consider the equilibrium $\left({\bar{p}}^{\left(1\right)},{\bar{p}}^{\left(2\right)}\right)=\left(1,0\right)$; the calculation for (0, 1) is completely analogous.

To get rid of the min in Eqs (30) and (31) we focus in addition on the case where market 1 is saturated with sellers:

$$\begin{array}{c} \frac{f_{1}V\left(\text{b},1\right)}{V(\text{a},1)}<1 \end{array}$$ (39)

As a consequence the min term disappears from the market conditions:

$$\begin{array}{c} M\left(\text{b},1,f_{m}\right)=M\left(\text{a},2,f_{1}\right)=1 \end{array}$$ (40)

$$\begin{array}{c} M\left(\text{a},1,f_{m}\right)=M\left(\text{b},2,f_{2}\right)=\frac{f_{1}V\left(\text{b},1\right)}{V(\text{a},1)} \end{array}$$ (41)

Here the equality between $M(\text{a},1,f_{1})$ and $M(\text{b},2,f_{2})$ comes from the symmetry of the parameters. Because $\left({\bar{p}}^{\left(1\right)},{\bar{p}}^{\left(2\right)}\right)=\left(1,0\right)$, all agents from class 1 go to market 1 and so the buyer-to-seller ratios *f*_*m*_ from (4) are simple to express in terms of *p*_b_:

$$\begin{array}{c} f_{1}=\frac{1}{f_{2}}=\frac{p_{\text{b}}}{1-p_{\text{b}}} \end{array}$$ (42)

The payoffs at the two markets for traders from class 1 simplify accordingly:

$$\begin{array}{c} P_{1}^{\left(1\right)}\left(f_{1}\right)=p_{\text{b}}V\left(\text{b},1\right)\left\langle S_{\text{b,1}}\right\rangle +\left(1-p_{\text{b}}\right)V\left(\text{a},1\right)\left[\frac{p_{\text{b}}}{1-p_{\text{b}}}\frac{V(\text{b},1)}{V(\text{a},1)}\right]\left\langle S_{\text{a,1}}\right\rangle \end{array}$$ (43)

$$\begin{array}{c} P_{2}^{\left(1\right)}\left(f_{1}\right)=\left(1-p_{\text{b}}\right)V\left(\text{a},2\right)\left\langle S_{\text{a,2}}\right\rangle +p_{\text{b}}V\left(\text{b},2\right)\left[\frac{p_{\text{b}}}{1-p_{\text{b}}}\frac{V(\text{b},1)}{V(\text{a},1)}\right]\left\langle S_{\text{b,2}}\right\rangle \end{array}$$ (44)

The factors in brackets are the matching probabilities from (41), from which V(a,1) cancels in the first equation and similarly (by symmetry) V(a,1)=V(b,2) in the second.

Our assumed equilibrium $\left({\bar{p}}^{\left(1\right)},{\bar{p}}^{\left(2\right)}\right)=\left(1,0\right)$ will be a Nash equilibrium if the payoff at market 1 is higher than at market 2 for players from class 1. (By symmetry, the payoff relation is then reversed for players in class 2.) From the explicit payoff expressions above, this condition can be re-arranged into

$$\begin{array}{ccc} 0 & \leq & p_{\text{b}}^{2}\left(-\left\langle S_{\text{a},2}\right\rangle V\left(\text{a},2\right)-\left\langle S_{\text{b},1}\right\rangle V\left(\text{b},1\right)-\left\langle S_{\text{a},1}\right\rangle V\left(\text{b},1\right)-\left\langle S_{\text{b},2}\right\rangle V\left(\text{a},2\right)\right)\\ & & +p_{\text{b}}\left(\left\langle S_{\text{b},1}\right\rangle V\left(\text{b},1\right)+2\left\langle S_{\text{a},2}\right\rangle V\left(\text{a},2\right)+\left\langle S_{\text{a,1}}\right\rangle V\left(\text{b},1\right)\right\rangle )-\left\langle S_{\text{a},2}\right\rangle V\left(\text{a},2\right) \end{array}$$ (45)

**Fig 15:**
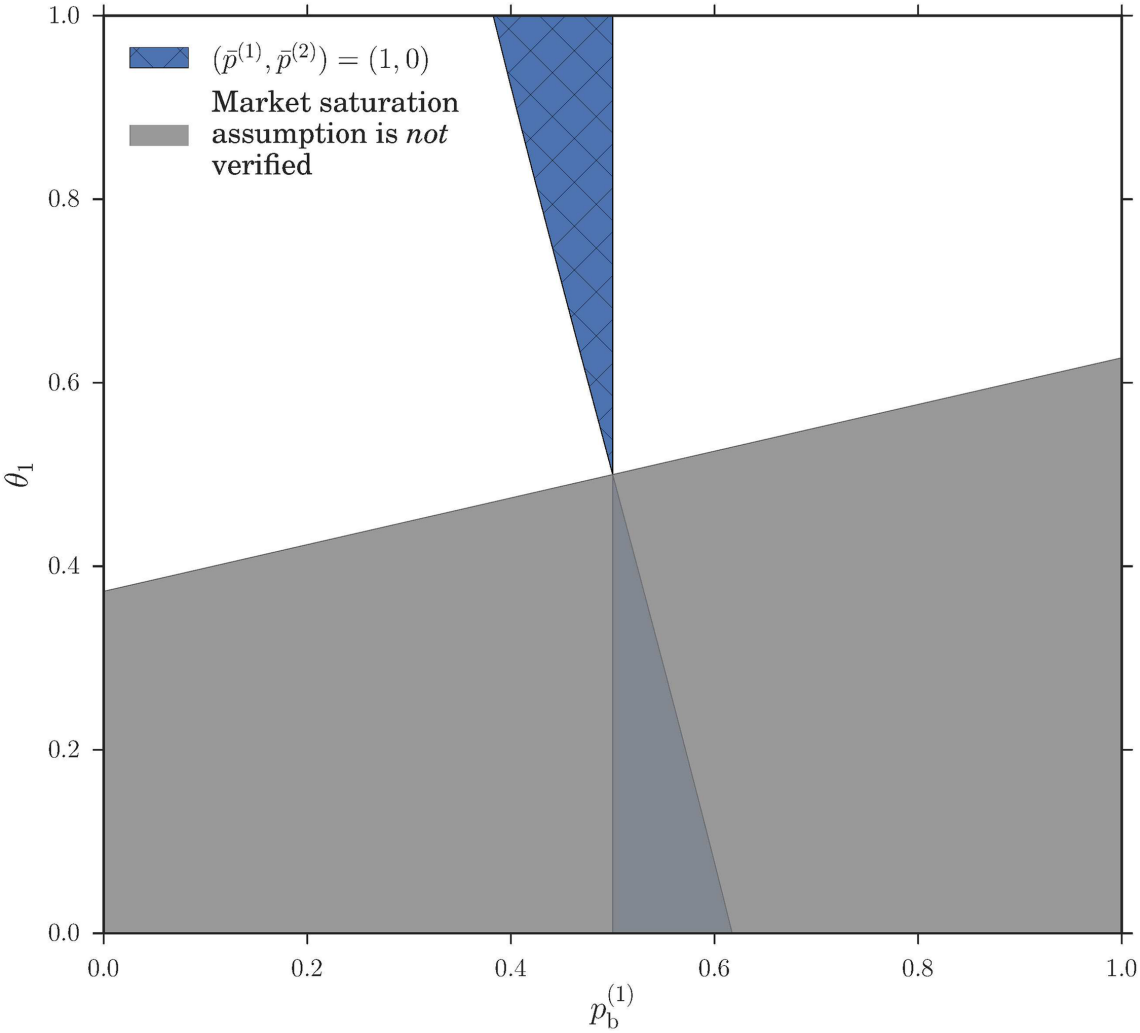
Analytic determination of boundaries for the zone where a homogeneous Nash equilibrium exists where players from the two classes choose different markets. Within the blue regions the payoff inequality (45) is satisfied. The region shaded grey is ruled out by the assumption of market 1 being saturated with sellers.

For given *θ*_1_ all coefficients in this quadratic equation are known so the phase boundaries can be obtained directly as its roots. We plotted these roots in Fig 15; note that the boundaries are close to linear but not exactly so. One has to check a posteriori that the assumption (39) of market 1 being saturated with sellers is valid, which rules out the bottom “cone” in the figure.

The remainder of the phase diagram in Fig 4 is obtained by the analogous calculation under the assumption that market 1 is saturated with buyers rather than sellers, which yields the bottom “cone” in Fig 15 and by finally repeating the overall reasoning for the Nash equilibrium $\left({\bar{p}}^{\left(1\right)},{\bar{p}}^{\left(2\right)}\right)=\left(0,1\right)$.

### Kramers-Moyal expansion

Here we provide the coefficients of the Kramers-Moyal expansion for traders with fixed buy-sell preference, given fictitious play coefficient *α* and intensity of choice *β*. The truncation of the Kramers-Moyal expansion at the second order gives the Fokker-Planck equation for the time evolution of the attraction distributions:

$$\begin{array}{ccc} \partial_{t}P\left(A^{\left(c\right)},t\right) & = & -\sum_{1\leq m\leq 2}\partial_{A_{m}^{\left(c\right)}}\left[\mu_{m}^{\left(c\right)}\left(A^{\left(c\right)},{\bar{p}}^{\left(1\right)},{\bar{p}}^{\left(2\right)}\right)P\left(A^{\left(c\right)},t\right)\right]\\ & & +\frac{r}{2}\sum_{1\leq m,m^{\prime }\leq 2}\partial_{A_{m}^{\left(c\right)}A_{m^{\prime }}^{\left(c\right)}}^{2}\left[\Sigma_{mm^{\prime }}^{\left(c\right)}\left(A^{\left(c\right)},{\bar{p}}^{\left(1\right)},{\bar{p}}^{\left(2\right)}\right)P\left(A^{\left(c\right)},t\right)\right] \end{array}$$ (46)

To lighten the notation we will in the following drop the superscript (*c*) indicating the class of an agent and also suppress the dependence on the aggregates ${\bar{p}}^{\left(1\right)},{\bar{p}}^{\left(2\right)}$, which are in general time-dependent via Eq (17).

In the above expansion time has been rescaled as *t* = *rn*, where *n* is the number of trading rounds. The time interval Δ*t* = *r* then features in the normalization of the drift and diffusion matrix, which are determined as the first and second order jump moments:

$$\begin{array}{c} \mu =\frac{1}{r}\left\langle \Delta A\right\rangle ,\;r\Sigma =\frac{1}{r}\left\langle \Delta A\;\Delta A^{\text{T}}\right\rangle \end{array}$$ (47)

where Δ**A** = **A**(*n* + 1) − **A**(*n*) is the change in the agent’s attraction vector in one training round and the T superscript indicates vector transpose. Writing Δ**A** explicitly from (2) then gives for the drift term:

$$\begin{array}{c} \mu_{1}\left(A\right)=\left[P_{1}\left(f_{1}\right)-A_{1}\right]\sigma_{\beta }\left(A_{1}-A_{2}\right)-\alpha A_{1}\sigma_{\beta }\left(A_{2}-A_{1}\right) \end{array}$$ (48)

$$\begin{array}{c} \mu_{2}\left(A\right)=\left[P_{2}\left(f_{2}\right)-A_{2}\right]\sigma_{\beta }\left(A_{2}-A_{1}\right)-\alpha A_{2}\sigma_{\beta }\left(A_{1}-A_{2}\right) \end{array}$$ (49)

In the diffusion term Σ_*ij*_ the second order moments of the score distribution also feature, as follows:

$$\begin{array}{c} \Sigma_{11}\left(A\right)=\left[Q_{1}\left(f_{1}\right)-2A_{1}P_{1}\left(f_{1}\right)+{A_{1}}^{2}\right]\sigma_{\beta }\left(A_{1}-A_{2}\right)+\alpha^{2}{A_{1}}^{2}\sigma_{\beta }\left(A_{2}-A_{1}\right) \end{array}$$ (50)

$$\begin{array}{c} \Sigma_{22}\left(A\right)=\left[Q_{2}\left(f_{2}\right)-2A_{2}P_{2}\left(f_{2}\right)+{A_{2}}^{2}\right]\sigma_{\beta }\left(A_{2}-A_{1}\right)+\alpha^{2}{A_{2}}^{2}\sigma_{\beta }\left(A_{1}-A_{2}\right) \end{array}$$ (51)

$$\begin{array}{c} \Sigma_{12}\left(A\right)=-\alpha \left[P_{1}\left(f_{1}\right)A_{2}\sigma_{\beta }\left(A_{1}-A_{2}\right)+P_{2}\left(f_{2}\right)A_{1}\sigma_{\beta }\left(A_{2}-A_{1}\right)-A_{1}A_{2}\right] \end{array}$$ (52)

$$\begin{array}{c} \Sigma_{21}\left(A\right)=\Sigma_{12}\left(A\right) \end{array}$$ (53)

### Fixed points of single agent dynamics

We show here generally that the single agent dynamics can have up to five fixed points, which can be determined from a single nonlinear equation. As before we drop the superscript (*c*) for the agent class. The aggregates and hence the expected payoffs $P_{1}$, $P_{2}$ are fixed.

Fixed points are found from the condition that the drift (48 and 49) must vanish:

$$\begin{array}{c} 0=\left(P_{1}-A_{1}\right)\sigma_{\beta }\left(A_{1}-A_{2}\right)-\alpha A_{1}\sigma_{\beta }\left(A_{2}-A_{1}\right) \end{array}$$ (54)

$$\begin{array}{c} 0=\left(P_{2}-A_{2}\right)\sigma_{\beta }\left(A_{2}-A_{1}\right)-\alpha A_{2}\sigma_{\beta }\left(A_{1}-A_{2}\right) \end{array}$$ (55)

Writing Δ = *A*_1_ − *A*_2_ and using *σ*_*β*_(*A*_2_ − *A*_1_) = 1 − *σ*_*β*_(Δ), one can express *A*_1_ and *A*_2_ in terms of Δ:

$$\begin{array}{ccc} A_{1} & = & \frac{P_{1}\sigma_{\beta }\left(\Delta \right)}{\sigma_{\beta }\left(\Delta \right)+\alpha \left[1-\sigma_{\beta }\left(\Delta \right)\right]}=\frac{P_{1}}{1+\alpha \exp(-\beta \Delta )} \end{array}$$ (56)

$$\begin{array}{ccc} A_{2} & = & \frac{P_{2}\left[1-\sigma_{\beta }\left(\Delta \right)\right]}{1-\sigma_{\beta }\left(\Delta \right)+\alpha \sigma_{\beta }\left(\Delta \right)}=\frac{P_{2}}{1+\alpha \exp(\beta \Delta )} \end{array}$$ (57)

Taking the difference gives a single equation for Δ, which takes a suggestive form if we write *α* = exp(−*aβ*):

$$\begin{array}{c} \Delta =\frac{P_{1}}{1+\exp(-\beta (\Delta +a))}-\frac{P_{2}}{1+\exp(\beta (\Delta -a))} \end{array}$$ (58)

**Fig 16:**
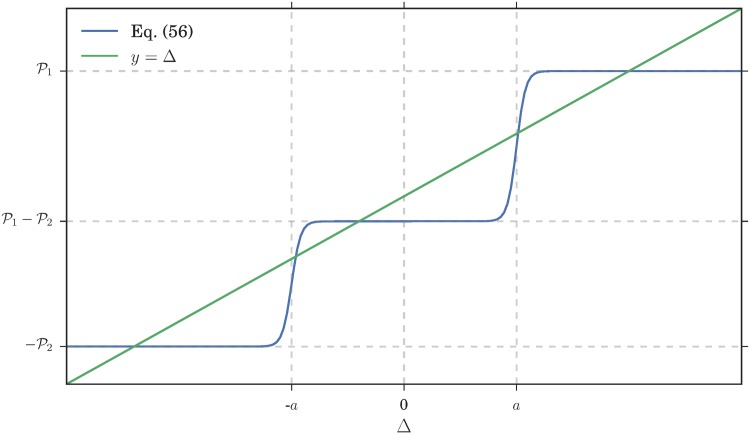
Sketch of the right hand side of the fixed point Eq (58) for Δ.

The solutions of this equation, and hence the single agent fixed points, can be obtained graphically by intersecting a straight line (the l.h.s. of Eq (58)) with the function of Δ on the r.h.s. This function has a simple shape as it is the sum of two sigmoids, one increasing from zero to $P_{1}$ around Δ = −*a* and the other increasing from $-P_{2}$ to zero around Δ = *a*. From the resulting shape, shown in Fig 16, at most five intersections with the diagonal can occur.

We are most interested in the limit of large intensity of choice *β*, where the sigmoids become step functions. For small *α*, *i.e*. large *a*, the only solution is then $\Delta =P_{1}-P_{2}$. As *α* is increased and hence *a* is decreased, the sigmoidal steps move closer to the origin, each creating an additional pair of solutions when *a* equals the relevant payoff (see Fig 16). For large *β*, one therefore has as transition from one to three (two stable, one unstable) fixed points at

$$\begin{array}{c} \alpha ∼\exp(-\max\left(P_{1},P_{2}\right)\beta ) \end{array}$$ (59)

and from three to five (three stable, two unstable) fixed points at

$$\begin{array}{c} \alpha ∼\exp(-\min\left(P_{1},P_{2}\right)\beta ) \end{array}$$ (60)

At finite *β* the fixed points are shifted away from Δ = ±*a* and this would give corrections to *a* of order 1/*β*, which would in turn determine the prefactors of the above scalings. Note that as *a* decreases further, the two sigmoidal ramps will eventually overlap when *a* is of order 1/*β*, signalling a transition back to three (two stable) fixed points.

We show in Fig 5 that the scaling of the above *α*-values, taken at equal payoffs $P_{1}=P_{2}$ as is relevant for Nash equilibria, also gives a good account of the variation with *β* of *α*_*c*_ and $\alpha_{c}^{\prime }$. This suggests that the *α*-values where new fixed points appear, and where they contribute as peaks with weights of order unity to the steady state distribution, are relatively close, maybe only within a constant prefactor of each other.

## Conclusion

In this paper we studied a minimal model of agents choosing between two double auction markets, which is a special case of a large aggregative game. Previous work studying a form of reinforcement learning inspired by EWA (experience weighted attraction) learning in this system had found segregation, where a group of identical agents becomes heterogeneous by separating into sub-groups adopting different behaviours. We first asked the question of whether this phenomenon has an analogue in the Nash equilibria of the corresponding game, where—in contrast to the reinforcement learning dynamics—agents have full information about their expected payoffs.

In a game theoretical analysis we addressed this question within a setup where there are two classes of agents that typically buy and sell, respectively. We showed that two *aggregate* quantities, namely, the fraction of agents from each class choosing the first market, are sufficient to assess whether a distribution of strategies, *i.e*. market preferences, across the agents in each class is a Nash equilibrium or not. This allowed us to classify the Nash equilibria, according to the type of strategies played by the agents (pure or mixed) and according to the distribution of strategies being homogeneous (the entire class population plays the same strategy) or heterogeneous (the population is divided into subpopulations playing different strategies). The model parameters for which each of these Nash equilibria exists are summarized in Fig 4. A key conclusion is that there are regions of heterogeneous equilibria: these are the equilibrium analogues of dynamical spontaneous emergence of preferences heterogeneity as observed previously.

This answer to our first question had to be qualified, however, because there is in general an infinity of strategy distributions consistent with a given pair of aggregate values. The Nash equilibrium analysis can therefore only identify equilibria as *potentially* heterogeneous but leaves open the nature of the actual strategy distribution, which could be homogeneous mixed, heterogeneous pure or heterogeneous mixed. We therefore asked a second question of whether reinforcement learning dynamics, which we chose as a variation of EWA learning, can resolve this ambiguity, by identifying which Nash equilibria can be reached dynamically. We first argued that steady states of our variation of reinforcement learning should be Nash equilibria in the limit of perfect fictitious play (*α* → 0), long agent memory (*r* → 0) and best response (*β* → ∞). Non-trivially, however, this joint limit can be taken in several ways, as shown in the phase diagram in Fig 5: depending on how the point (*α*, 1/*β*) = (0, 0) is approached, a small number of different limiting steady states of our reinforcement learning dynamics can result as sketched in Fig 3. These include a homogeneous mixed state, where all agents within a class randomize between markets in the same way, and a heterogeneous pure equilibrium, where agents separate into two groups, each choosing a market deterministically. Along with these standard types of Nash equilibria, however, we also found a *heterogeneous mixed* steady state, where the agents do split into groups but not all groups play deterministically. In fact, at the boundary between the latter two types of steady states (denoted $\alpha =\alpha_{\text{c}}^{\prime }$ in our analysis) it is possible to generate equilibria where *three* groups of agents appear within each class.

Technically what made our theoretical analysis of the heterogeneous steady states possible was the use of Freidlin-Wentzell theory, which is the tool of choice for studying the behavior of dynamical systems subject to weak noise, here arising from the limit *r* → 0. We also compared the theoretical results to multi-agent simulations for *r* > 0, finding good qualitative agreement.

While we focused our analysis on the study of the minimal model of choice between double auction market presented in the model section, our methods could be applied fruitfully also to the study of reinforcement learning in other types of aggregative games such as the Cournot model [36]. It would be particularly interesting to see whether also here dynamical considerations single out particular Nash equilibria, including ones with the novel heterogeneous mixed character that we found in our system.

At a technical level, future work could look more closely at the limit of large intensity of choice *β* required to realize Nash equilibria as dynamical steady states. We approached this limit numerically, finding good agreement with theoretical predictions already for relatively modest *β*. An interesting challenge would be to take the full *β* → ∞ limit in closed form within the analysis: preliminary work suggests that the large deviation analysis then becomes rather intricate, hence we leave this aspect for future work.
